# BRIGHTLIGHT researchers as ‘dramaturgs’: creating *There is a Light* from complex research data

**DOI:** 10.1186/s40900-020-00222-5

**Published:** 2020-08-10

**Authors:** Rachel M. Taylor, Brian Lobel, Keisha Thompson, Adura Onashile, Mark Croasdale, Nathaniel Hall, Faith Gibson, Ana Martins, David Wright, Sue Morgan, Jeremy S. Whelan, Lorna A. Fern

**Affiliations:** 1 Centre for Nurse, Midwife and Allied Health Profession Led Research (CNMAR), University College London Hospitals NHS Foundation Trust, London, UK; 2 Rose Bruford College, Sidcup, UK; 3 The Royal Central School of Speech and Drama, London, UK; 4 Contact Young Company, Contact Theatre, Manchester, UK; 5 School of Health Sciences, Faculty of Health and Medical Sciences, Kate Granger Building, University of Surrey, Guildford, UK; 6 Centre for Outcomes and Experience Research in Children’s Health, Illness and Disability (ORCHID), Great Ormond Street Hospital for Children NHS Foundation Trust, London, UK; 7 Cancer Division, University College London Hospitals NHS Foundation Trust, London, UK; 8 Top Floor Palatine Treatment Centre, The Christie Hospital, Manchester, UK; 9 Teenage and Young Adult Cancer Unit, Leeds Teaching Hospitals NHS Trust, Leeds, UK

**Keywords:** Adolescents, Young adults, Teenagers, Cancer, Dissemination, BRIGHTLIGHT, Patient and public involvement, Theatre, Art

## Abstract

**Background:**

BRIGHTLIGHT is a national evaluation of cancer services for young people aged 13–24 years in England. It is a mixed methods study with six interlinked studies aiming to answer the question: do specialist cancer services for teenagers and young adults add value? http://www.brightlightstudy.com/. Young people have been integral to study development and management, working as co-researchers, consultants and collaborators throughout. We aimed to share results in a way that was meaningful to young people, the public, and multidisciplinary professionals. This paper reports the development of ‘*There is a Light: BRIGHTLIGHT’,* a theatrical interpretation of study results by young people, and offers insight into the impact on the cast, researchers and audiences.

**Methods:**

The BRIGHTLIGHT team collaborated with Contact Young Company, a youth theatre group in Manchester. Twenty members of Contact Young Company and four young people with cancer worked together over an eight-week period during which BRIGHTLIGHT results were shared along with explanations of cancer, healthcare policy and models of care in interactive workshops. Through their interpretation, the cast developed the script for the performance. The impact of the process and performance on the cast was evaluated through video diaries. The research team completed reflective diaries and audiences completed a survey.

**Results:**

*‘There is a Light’* contained five acts and lasted just over an hour. It played 11 performances in six cities in the United Kingdom, to approximately 1377 people. After nine performances, a 30-min talk-back between members of the cast, creative team, an expert healthcare professional, and the audience was conducted, which was attended by at least half the audience. Analysis of cast diaries identified six themes: initial anxieties; personal development; connections; cancer in young people; personal impact; interacting with professionals. The cast developed strong trusting relationships with the team. Professionals stated they felt part of the process rather than sitting on the periphery sharing results. Both professional and lay audiences described the performance as meaningful and understandable. Feedback was particularly positive from those who had experienced cancer themselves.

**Conclusions:**

Using theatre to present research enabled BRIGHTLIGHT results to be accessible to a larger, more diverse audience.

## Plain English summary

BRIGHTLIGHT is a research study examining the experience of having cancer as a young person and if there are benefits to young people of providing TYA-specific cancer care. The study was designed with young people, who worked as co-researchers, collaborators and consultants. BRIGHTLIGHT has recruited over 1000 young people. We wanted to create a meaningful and accessible way to disseminate emerging BRIGHTLIGHT results with young people as partners.

We worked with a young theatre group, four young people with cancer, clinicians and theatre directors to create *‘There is a light’*, an hour-long arts-based performance based on BRIGHTLIGHT results. Twenty-four young people attended a series of workshops and rehearsals over eight-weeks where BRIGHTLIGHT results were shared along with explanations of healthcare policy, cancer and delivery of care. The young people and professionals formed strong, trusting relationships due to the sensitive nature of the topic. The cast learned about cancer and gained new skills as actors, acquired cancer and healthcare policy knowledge and felt their ability to talk with young people who had experienced cancer was enhanced.

The performance toured public, professional and patient events, reaching 1377 people, with 11 performances in six cities. Audience feedback agreed that theatre was an effective way to disseminate study results in terms of increasing cancer knowledge and awareness however not all audience members realised the play was based on real study results. This paper describes development of the performance and offers insight into the impact on the researchers, the cast, and audiences.

## Introduction

*Within the wider taboos associated with illness in general and cancer in particular, voices of resistance, provocation, humour and empowerment can be drowned out or forgotten. ‘There is a light: BRIGHTLIGHT is our attempt to bring those voices to the forefront’*

*Adura Onashille (Director)* [1]

Knowledge translation of health research is broadly defined as the method for closing the gaps between knowledge and practice [2]. Translation requires targeting different stakeholders to assign clinical meaning to data and to translate research for use at the bedside. The first step is dissemination, a process through which study results are shared, and key messages for practice discerned. Traditionally, dissemination has been limited to peer-reviewed publications and conference presentations, but more non-traditional methods, such as social media are expanding rapidly [3]. Despite expansion of non-traditional methods of dissemination, text and jargon heavy results are commonplace which are often not accessible to all professionals and importantly the patients who took part in the study and those who may benefit from study results in the future. Consequently, effective and multiple modes of communication is needed, between those who produce the knowledge (researchers) and the users of knowledge (healthcare teams, policy makers, patients and the public).

However, knowledge alone is not enough to inform and influence practice. Traditional and more ‘linear strategies’ for sharing knowledge have been described as inadequate and more circular methods have been advocated [4]. Indeed, Reiger and Schultz noted that healthcare professional knowledge predominantly came from interactions with colleagues, and therefore using methods that will make results memorable could be a first step in getting evidence into practice [4]. If we want to reduce the time lag in applied health translational research, currently estimated to be about 17 years, then as researchers we need to be creative in the way we share research findings, in order to take new knowledge ‘from the bench to the patients’ bedside’ [5].

Knowledge translation strategies are overt activities that facilitate or encourage the use of research to achieve clinical practice change [6]. More recent strategies have included evidence briefs, practice guidelines, toolkits and plain language summaries. Increasingly, healthcare researchers are also using arts-based strategies, although much less is known about how this is used. Certainly, in the United Kingdom (UK) there has been an increasing interest in the link between arts and health. The Department of Health reported in 2007 on a working group to review their role in promoting arts and health [7], and in 2014 an All-Party Parliamentary Group on Arts, Health and Wellbeing was formed to “improve awareness of the benefits that the arts can bring to health and wellbeing, and to stimulate progress towards making these benefits a reality all across the country” [8]. Their subsequent report concluded that the arts had a positive impact on health, had the ability to help patients manage health challenges and subsequently save the National Health Service (NHS) money [8]. The focus of these reports has been on the benefits of arts to health but did not explore the relationship between the role of the arts and health research. The arts are recognised as an important medium for successfully achieving participant engagement [9], thereby making the arts an attractive option for disseminating research to generate greater impact than an academic conference presentation or manuscript [10].

Arts-based knowledge translation strategies have been broadly grouped into three categories, visual (photographs, drawings, art exhibition), literary (poetry), or performance (theatre, narrative based-arts) [11]. These are used to translate key, educative messages to broader audiences. Theatre has emerged as a key medium [12]. While four genres of performance have been described, two dominate current literature reporting the use of theatre in heath research [13]: ethnodrama and theatrical research-based performance. Ethnodrama, remains true to informant data so uses ethnographic observation and participant transcripts to create the script and involves the research team throughout its development [13, 14]. Conversely, theatrical research-based theatre is informed by the research but does not necessarily utilise data. The cast draw on their own experiences to interpret results, often without input from the research team [13, 15]. Ethnodrama requires qualitative data to inform the content of the script and therefore is not suited to quantitative and mixed methods studies where large datasets inform a substantial aspect of the results.

*‘There is a Light: BRIGHTLIGHT’* is a research-based theatrical performance created by a youth theatre group. The Cast consisted of young people including young people with a previous cancer diagnosis, the performance depicts their interpretation of results from a programme of research. Interpretations can end up quite far from the original source materials [14, 15]. However, *There is a Light* uniquely saw the research team behind the study as *dramaturgs*, a theatre term for a professional (not unlike a director) who is responsible for how an audience understands or interprets a given theatrical story, plot or stage picture [10]. Theatre may be an effective knowledge translation strategy because it is a commonplace and culturally acceptable activity in many countries and communities. The aim of the performance was to increase the accessibility and reach of BRIGHTLIGHT study results. Recognising that utilising our traditional dissemination methods would confine our results to peer reviewed manuscripts and professional conference presentations. This would exclude many of the multidisciplinary team members involved in the care of young people who do not access clinical/medical conferences. This includes but is not limited to youth support coordinators, social workers, third party representatives, non-research nurses, physiotherapists, complementary therapists, administrators and notably at a time with increasing financial pressures, nurses and doctors who cannot access study leave or funds to attend conferences. Critically, limiting dissemination to traditional methods would exclude young people themselves, their friends and families from finding out about the study results and the experience of being a young person with cancer. The aim of this paper is to describe the process of creating the theatrical performance, ‘*There is a Light: BRIGHTLIGHT’,* from a programme of research, and the evaluation of the impact of the subsequent performance on the audience, cast and research team. For clarity, Table 1 summarises the aims of BRIGHTLIGHT, the aims of the performance and the aims of this paper.

**Table 1:** Summary of the aims of BRIGHTLIGHT, the performance and this paper

**Aims of BRIGHTLIGHT**	To evaluate specialist care for young people with cancer in England. Evaluating whether treating young people in a specialist cancer unit with professionals who are expert at treating cancer and expert at treating young people impacts on young people’s quality of life, survival and experience of being a young person with cancer
**Aims of performance**	To increase accessibility and reach of the BRIGHTLIGHT results. Particularly to enable those impacted most by cancer in young people, young people themselves, the opportunity to see the results in an understandable way. We also sought to allow more multidisciplinary professionals access to the results but not confining dissemination to traditional peer reviewed articles and conference presentations which are often inaccessible to many members of the multidisciplinary team involved in the care of young people. We aimed to ensure young people were at the heart of the production allowing them to interpret the emerging BRIGHTLIGHT results as they felt appropriate.
**Aim of this paper**	Describe the process of creating the theatrical performance, ‘*There is a Light: BRIGHTLIGHT’*, from a programme of research, and the evaluation of the impact of the subsequent performance on the audience, cast and research team.

## The context: what is BRIGHTLIGHT?

BRIGHTLIGHT is a National Institute for Health Research (NIHR) funded programme of research (grant reference RP-PG-1209-10013), the study aims to evaluate cancer services for young people aged 13–24 years in England [16]. A cancer diagnosis during adolescence and young adulthood has an acute impact on a critical and complex stage of life development, disrupting physical health, social and educational goals as well as psychological wellbeing [17]. Furthermore, young people experience a unique spectrum of cancer types, which are distinct from those affecting younger children and older adults.

While potentially curable for many young people, there is evidence that outcomes for some cancers have not improved in line with those achieved for children and older adults [18]. However, there are advantages to society from the successful treatment of young people with cancer through the prolonged fulfilment of their contribution in employment and other societal impacts [19]. Consequently, guidance was published in 2005 by the National Institute for Health and Care Excellence (NICE), which recommended that young people aged 15–18 years would be cared for in a specialist teenage and young adult (TYA) unit and those aged 19–24 years receive ‘unhindered access’ to specialist care [20]. To accommodate this, 13 TYA Principal Treatment Centres were developed across England, which then hosted these specialist units creating a hub of excellence and expertise.

In contrast to the recommendations for children’s cancer services in the NICE guidance, which were based on considerable evidence, those for TYA were underpinned by limited evidence [20]. The BRIGHTLIGHT study was developed to provide this evidence, comprising of six interlinked research projects that collectively are designed to answer the question: Do specialist cancer services for TYA add value? The BRIGHTLIGHT studies include: an international e-Delphi study to define the core competencies required by healthcare professionals caring for young people with cancer [21]; a case study across 28 hospitals in England examining the culture of TYA cancer care [22, 23]; the development of a metric from secondary analysis of NHS hospital admission data to quantify time spent in the Principal Treatment Centre [16]; establishing a cohort of 1114 young people newly diagnosed with cancer between 2012 and 2014 [16]; and administering a bespoke survey five times over 3 years from six to 36 months after diagnosis [24]; a survey of the unmet needs to caregivers of young people with cancer [25]; and a health economics evaluation of young people and caregivers’ out of pocket expenses and cost of specialist care to the NHS.

BRIGHTLIGHT was developed with young people, and the Young Advisory Panel (YAP) have been core members of the research team throughout the study [26]. In addition to the six BRIGHTLIGHT studies, additional work has been undertaken with the YAP exploring recruitment to research [27], participant retention [28] and sexuality, relationships and body image [29]. The collective evidence, including the BRIGHTLIGHT feasibility work up to January 2017, was used to inform the content of the performance ‘*There is a Light: BRIGHTLIGHT’*.

## Choosing performance as a mode of dissemination

There are many modes of public engagement and research dissemination, to ensure we selected the appropriate medium we consulted with the BL who was at the time, the Wellcome Trust Public Engagement Fellow. We provided a clear brief:

- We wanted to make BRIGHTLIGHT results more accessible
- We wanted to improve understanding amongst professionals and the public about what it was like to be a young person with cancer.
- We wanted to involve young people in the dissemination of BRIGHTLIGHT
- We wanted to include not just a professional medical audience but include the people who mattered most to BRIGHTLIGHT, young people themselves, their friends and families.
- We wanted professionals who do not read peer reviewed medical journals to access BRIGHTLIGHT results and for those who do not make it to conferences - an increasing number now due to funding difficulties. We wanted to include the multidisciplinary team members including social workers, youth support workers, research nurses, ward staff and third sector representatives who provide support for young people with cancer.

After a number of meetings with BL we chose theatre performance as the most suitable mode of dissemination and engaged with KT from Contact Young Company (see below). We created a proposal for ‘There is a light’ and submitted it to The Wellcome Trust Public Engagement call where it was peer reviewed and funded based on its appropriateness and rigour.

## The cast

Contact is an arts centre in Manchester. Contact Young Company (CYC) are an artist ensemble comprising of 20 young people aged 15–25 years who audition to be a member of the company for three performances. The CYC are recognised for addressing sensitive issues in a sympathetic but realistic way, for example, disability rights (*Ramping up*).

The aim for ‘*There is a Light’* was to also include four young people from either the YAP or participants in the BRIGHTLIGHT cohort to help the CYC contextualise the BRIGHTLIGHT results into real life experiences. However, the need to attend regular workshops and rehearsals in Manchester made this unfeasible for interested BRIGHTLIGHT participants who were out with the immediate Manchester area. As an alternative the BRIGHTLIGHT cohort participants who applied to be in the cast were offered the opportunity to attend workshops as experts. This ensured their perspective was included. Young people with a previous cancer diagnosis were identified locally with the assistance of the local youth support coordinator who advertised the opportunity; four auditioned and became members of the cast. Young people with cancer shared their cancer experience with young people who did not have cancer and assisted with the interpretation of study results.

## Developing the script

The script was devised by young people through learning about cancer in young people and interpreting BRIGHTLIGHT results. As opposed to the research being handed over to the cast as stand-alone data, a series of workshops were undertaken with the BRIGHTLIGHT research ‘*dramaturgs*’ (members of the research team, the cohort, YAP and healthcare professionals), who were integrated throughout the workshop process. This ensured the young cast of theatrical interpreters had opportunities to ask questions, talk through jargon and day-to-day experience, and respond critically and provocatively to the research. Additional theatrical technique training was delivered by the Lead Artist (NH). The twice-weekly workshops commenced on 12th January 2017 and were held over 5 weeks. Each week one workshop was focused on BRIGHTLIGHT results and interpretation of the data which was facilitated by BL. These workshops were attended by two professionals, for consistency LF or RT attended all workshops along with one other member of the BRIGHTLIGHT research team or a clinical expert. This was to allow young people the opportunity to ask questions about the research or clinical care for young people. The cast were also offered the opportunity to visit the local Teenage and Young Adult Cancer Unit to experience the environment of care. The second weekly workshop was focussed on acting skills, voice training and choreography. Excerpts from workshops and rehearsals can be viewed in the BRIGHTLIGHT documentary here: https://vimeo.com/238045094.

**Fig. 1:**
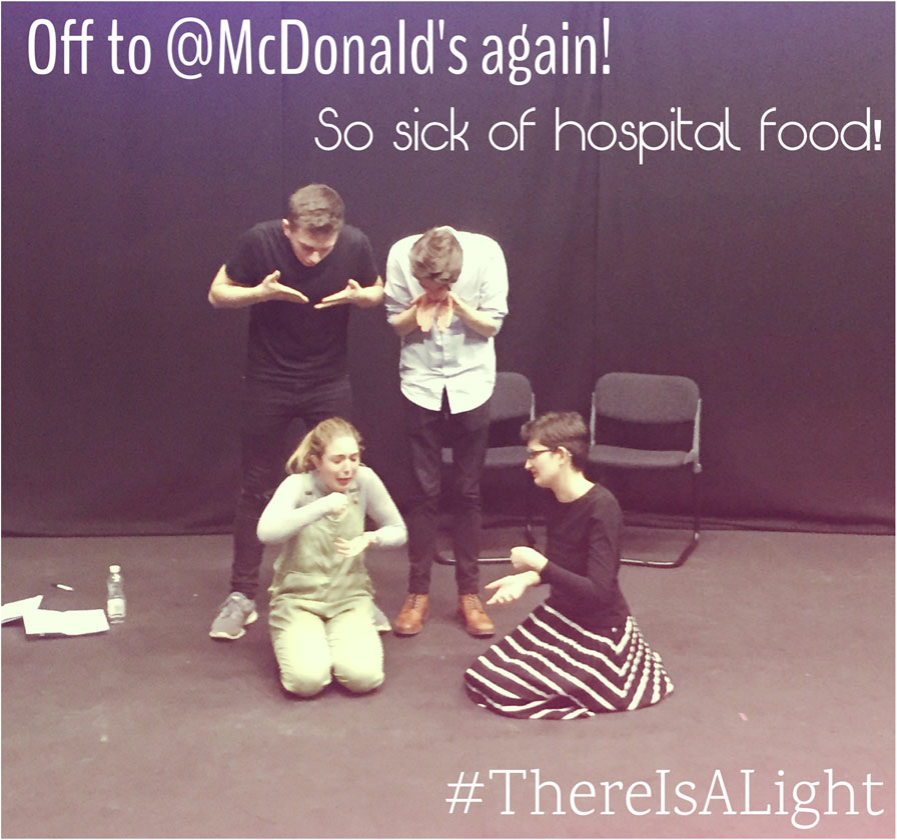
An example of a social media posting created by the cast to relate a complaint young people have of hospitals

The cast were supported by the Project Director (BL), who facilitated the delivery of key messages from BRIGHTLIGHT, which were divided into weekly themes: BRIGHTLIGHT study-related information; patient perspective; caregiver perspective; doctor perspective; and nurse perspective. A briefing document was provided to the Project Director containing emerging results (Table 2). He then translated the results into creative activities for the CYC to encourage an embodied and comprehensive understanding of the material, to help them interpret and translate results into ideas for a performance. For example, young people were asked to make social media postings related to common complaints young people had of hospitals, so they had translated what they read about the complaint into a visual interpretation of it (see Fig. 1).

**Table 2:** Summary of the content of the briefing document

• Summary of the study design and methods in each of the six research projects	
• Challenges in involving young people in research:	
▪ Graphs of recruitment to the cohort [16]	
▪ Summary of the analysis of screening log data from the cohort^a^	
▪ Young people’s perspectives of having access to research [27]	
▪ Challenges healthcare professionals faced in recruiting to the cohort [30]	
• Delivery of information and communication [31]	
▪ Cohort wave 1 results: not being given time to decide about fertility preservation^a^	
▪ Cohort wave 1 results: how young people found out that they had cancer^a^	
• Empowering young people gives you information you would not have thought to ask [31]	
• The continuing impact of cancer after treatment has ended and young people are living beyond their diagnosis [31]	
• The impact of a cancer diagnosis on carers [25]^a^	
• Young people’s reported experience of being treated on an adult, children or TYA specialist cancer ward [31]^a^	
• The environment of care [23]^a^	
• Cohort wave 1 results: the best and worst aspects of care^a^	
• Sexuality and cancer [29]	
• The value and importance of the TYA workforce [21]^a^	

^a^inclusion of unpublished data

The cast developed the content from their interpretations individually and in groups. Not all the ideas were developed further than an initial sharing with the group (see Table 3 as an example of the artistic representation of a young person’s pre-diagnosis experience that was not included in the final performance). Working together with the Director (AO) and Assistant Director (MC), young people agreed on the content of the performance. This drew on the cast’s personal experiences of illness and their strengths as performers.

**Table 3:** An example of text developed in the workshops not included in the final performance

There is a light in bright lighted Bertie.	
Bright lighted Bertie bulb was happy and bright.	
He lit up the room all through the night.	
But 1 day Bertie he felt a flicker.	
He thought if he ignored it, it would disappear.	
Loo Loo knew Bertie wasn’t right.	
“When people sit on me, they often have a sh****, sometimes a wee. Bertie please go and see the family GP.”	
Bertie thought hard and when it struck 12, he untwisted himself and rolled on to a shelf. He rolled his way down on to the landing where he found the stairs, so grand and disheartening.	
Bump, bump, bump went Bertie down the stairs! He finally arrived a little worse for wear.	
Bertie brushed it off and arrived at his GP, The family Grill Pan.	
“We’ve checked your records and you seem just fine, there is no reason for you to be faulty. Just drink more water.”	
Bertie decided to get a second opinion, to another GP this one in the garden.	
This GP, The Garden Pot found something the other hadn’t!	
“You need to get fixed before it’s too late. I can’t believe it’s gone this long, quick take a left after the gate” said the Pot.	
Bertie arrived at a shed, he was nervous, scared and full of dread.	
When Bertie entered there was a huge line!	
Full of other items ready for repair, there was a sense of urgency in the air.	
There was Sue the shoe, Brian the iron and Baz the battery.	
This comforted Bertie and he had a good chat.	
Brian the iron says, “We are friends that is that”.	
After such a long wait, Bertie had his procedure and after that it was about getting better. Bertie had his moments as it was tuff but his friends support was just enough.	
“I’m glad you are back Bertie it’s been pitch black in here! When Alison came in, she s*** on me ear!” Said Loo Loo.	
Now Bertie shines brighter than ever!	
With new friends in mind and getting better, Bertie feels as light as a feather.	
THE END!	

Devising and rehearsing 4-day-a-week intensive rehearsals, led by the Director, began on 13th February for 3 weeks, and the performance opened on 8th March 2017. Unlike many theatrical research-based performances [13, 15, 32–34], *There is a Light* had minimal input from the research team after the workshop/dramaturgy stage was completed. The development of the script was entrusted to the responsibility of the cast, with guidance from the creative team. Neither the research team nor the funder saw the performance until the opening night.

## The performance

The final script comprised of five overarching ‘acts’ taking the audience through the experience of having cancer as a young person from pre-diagnosis (diagnosis), treatment and care (after the shock), support (support) to cure (survival) or death (Table 4, Additional file 1). At the end of nine performances there was a 30-min talk back with some members of the cast, the creative team (KT, BL, MC) and supported by a healthcare professional or member of the BRIGHTLIGHT team (RT, JW, DW, LF, SM) who took questions from the audience.

**Table 4:** Scenes from *There is a Light: BRIGHTLIGHT*

Act	Scene
Diagnosis	Being told you have cancer Young people’s right to be involved in decisions Impact of finding out a parent has cancer Misdiagnosis 1 Reflection on having childhood cancer Misdiagnosis 2
After the shock	Young people excluded from research Undergoing treatment for cervical cancer Consultation with the GP Sensitive conversations with parents present NHS versus private healthcare Inequality in access to young person-specific cancer care Developing and maintaining relationships when you have cancer
Support	Young person-related services provided in specialist units and inequalities in accessing these Importance of carers
Death	Experience of losing a friend to cancer In memory of people the cast have lost
Survival	Reflections on being cured

*GP* General practitioner

## The evaluation

The performance was an hour long and opened the 2017 S!CK festival in Manchester (http://www.sickfestival.com/), performing for three nights then eight additional performances in six cities to a total of 1377 people. Performances were held for general theatre audiences which also included patients, their families, friends and professionals. Additionally, three conference performances specifically targeted nurse researchers who were mainly adult trained, multidisciplinary cancer specialists, and patients (Table 5). The audiences at the S!CK, Homegrown and Chrysalis festivals paid to attend these performances, whereas attendance was included in the programme at the three conference performances. Due to the heterogeneity of where the performance was shown it was not possible to receive exact demographic data from all audience participants.

**Table 5:** Performance schedule in 2017 for *There is a Light*

Date	Venue	Location	Audience	Attendance	Panel discussion
8-10th March	S!CK Festival	Contact Theatre, Manchester	General public	325	All three performance
12th March	S!CK Festival	Attenborough Centre for the Creative Arts, Brighton	General public	43	Yes
6th April	Royal College of Nursing (RCN) Annual International Nursing Research Conference	Oxford University, Oxford	Nurses	≈200	Yes
9th April	Homegrown Festival	Battersea Arts Centre, London	General public	100	Yes
7th November	National Cancer Research Institute (NCRI) Annual Conference	Arena and Convention Centre, Liverpool	Multidisciplinary cancer professionals and consumers (patients and carers)	160	Yes
17-19th November	Chrysalis Festival	Traverse Theatre, Edinburgh	General public	339	Matinee only
25th November	Find Your Sense of Tumour (FYSOT), patient residential event	St Georges Park, Birmingham	Young people with cancer	210	Yes

The evaluation of the performance was planned to consider the perspective of the audience, the cast, and the research team: each are considered in turn, drawing on both quantitative and qualitative data.

### Audience perspective

In line with our overall aim for ‘There is light’ we sought to evaluate if we had reached a large multidisciplinary professional audience and increased the accessibility of research results to the public. The evaluation of the audience reaction was undertaken through surveys and social media. A survey was developed based on evaluations of performances in previous studies [35]. This was administered in paper format on the opening night, online at the National Cancer Research Institute (NCRI) conference and in paper format at the Chrysalis festival. A total of 88 responses were returned (15%). Of those who responded most strongly agreed/agreed they learnt something new from the performance and had enhanced understanding of what it is like to be a young person with cancer. The majority also felt that using theatre was an effective way to educate people about cancer in young people and share research results (Table 6). This was supported by the free text comments noting the powerful messages that were conveyed in the performance, however the research element was not outwardly obvious (Table 7).

**Table 6:** *There is a Light* evaluation questionnaire results (*N* = 88)

Question	Number strongly agree/agree	Percentage
I have learned something new about what it is like to live as a young person with cancer	84	95
I have an enhanced understanding of what it is like to be a young person with cancer	85	97
I feel that the knowledge I have gained from this play will impact the way I interact with people with cancer	76	86
I think using research-based drama is an effective way of educating people about cancer in young people	86	98
Using drama helps me better understand the research findings	78	89
Given the opportunity, I would participate in further creative experiences related to research	78	89
Having seen the performance, I would be more likely to ask about research opportunities in my hospital	48	55
Having seen the performance, I would be more likely to ask about research opportunities at my GP surgery	43	49
Having seen the performance, I would be more likely to take part in research	56	64

**Table 7:** Examples of free text comments

“I have worked with children and young people with cancer for nearly 30 years and read many dry research reports. This was exciting, meaningful, understandable, I’m sure I will remember the detail for much longer than the hundreds of papers I’ve read before and I will talk about it. Thank you”	
“For a play about young people with cancer, unexpectedly life-affirming, warm, vital & real. From strength of responses in post-show, seemed there is a lack of platforms for researchers/medical experts, people affected by cancer to share experiences - thanks for going some way to changing that.”	
“Despite having considerable experience (academic, personal, and professional) in respect of cancer I still found the play to be informative, meaningful, at times sad, but overall a positive experience.”	
“An excellent performance from some wonderful talented young people. A powerful message, which was delivered with compassion and really understanding from the perspective of the young adult facing cancer.”	
“I loved the performance, but I think the fact that is was based on research was a little lost. Maybe I switched off for a second or two but although I thought it was fantastic, I didn’t come away with a strong understanding that it’s base had been research findings (as opposed to anecdotal experiences). As a cervical cancer survivor, I found the show particularly moving, and the girl who played that particular patient did so extremely well, especially exploring the issues of blame and shame around that specific cancer. Very good stuff indeed and I would love to see it in schools/colleges up and down the country. What struck me also was that teenage experience of diagnosis and treatment was not dissimilar to my own, the feelings and emotions were similar in an adult. A huge well done to all involved.”	
Free text from FYSOT event “inspirational and eye opening”	
“Brightlight (sic) was amazing! really memorable + relatable felt highly accurate”	
“Brightlight (sic) was absolutely incredible! Highlight of my weekend!”	
“They were very good, very beneficial and spreading awareness”	

The impact of the performance in motivating people to take part in research themselves was less clear, with less than half of respondents stating they would be more likely to ask about research opportunities and only 56% stating they would be more likely to take part in research after seeing the performance. Chrysalis conducted an in-house evaluation which was returned by 54 audience members (16%). There was agreement in all responses that the performance was moving and most agreed that it got them thinking about things differently (*n* = 49, 81%) and some aspects of the performance seemed relevant to their own lives (*n* = 44; 82%).

Young people’s evaluation of the performance at the patient event ‘Find Your Sense Of Tumour (FYSOT), scored 9.48 out of 10 and 10/74 (14%), and noted the performance was the best part of the weekend. Free text comments also expressed the relatability of the performance to their experience.

**Fig. 2:**
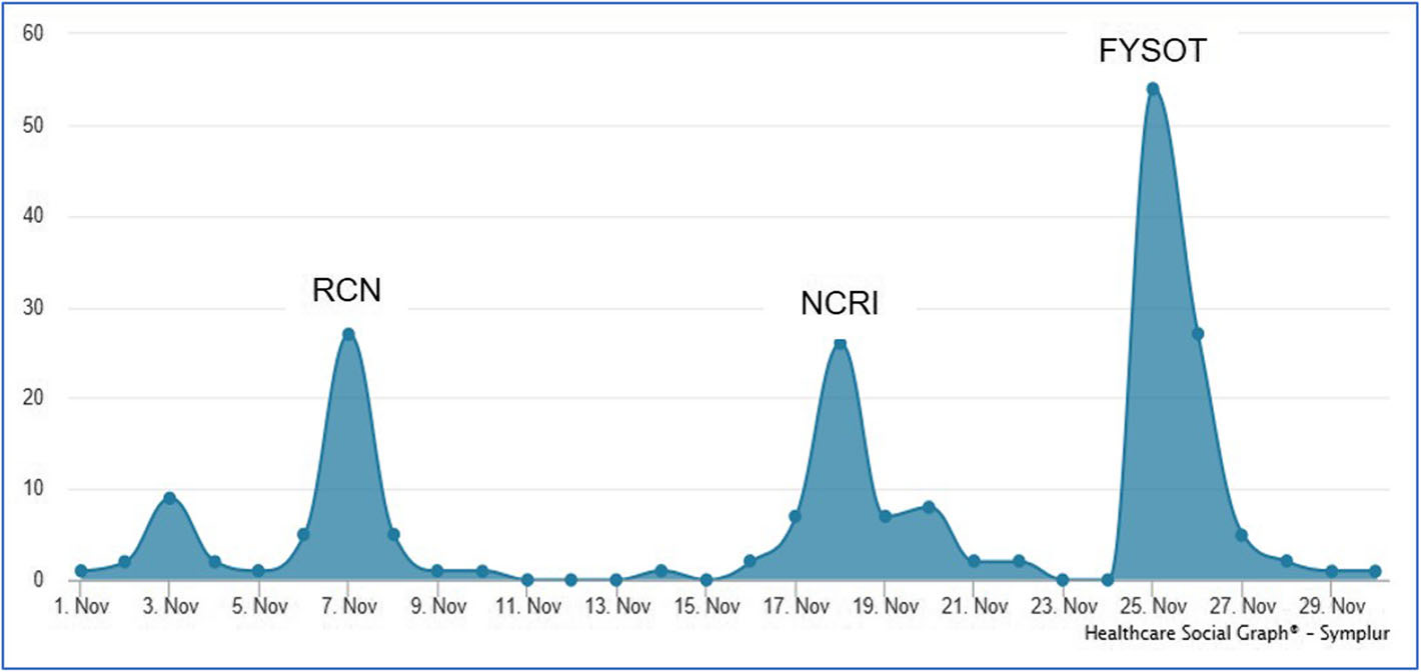
Twitter activity with #thereisalight November 2017

Evaluation of social media indicated there were 1192 Tweets using the hashtag #thereisalight, which peaked on the nights of the performance with the greatest twitter activity at the patient conference (Fig. 2). The performance was also reviewed by theatre critics, which were mostly positive (Table 8) and was short listed for the 2018 Manchester Theatre Award, Category ‘Youth Panel Award’. (https://northwestend.co.uk/index.php/homepage/news/2862-celebrating-the-best-of-2017-at-the-manchester-theatre-awards).

**Table 8:** Links to the external reviews of *There is a Light*

http://exeuntmagazine.com/reviews/review-sick-festival-manchester/	
https://youngwildandfibroblastic.com/2017/03/13/there-is-a-light-brightlight-issues-affecting-young-adults-with-cancer/	
http://www.jamesvarney.uk/revew-contact-young-company-light-brightlight/	
https://www.list.co.uk/article/98012-chrysalis-festival-there-is-a-light-brightlight-and-how-to-save-the-world-ish/	

### Cast perspective

The involvement of cast members with cancer into the CYC to participate in the development of the script, share their experience of having cancer and their interpretation of the research results, as well as take part in the performance was unusual.

We aimed to evaluate the experience of learning about BRIGHTLIGHT results, interpreting and contextualising with the young people who had a cancer experience and then translating this into a performance. The CYC were given video cameras and asked to record a weekly diary reflecting on the process. We aimed to capture their first impressions, what they expected to get out of participation and what they wanted the audience to get out of the performance. In subsequent weeks we asked them to reflect on what they liked that week, what they did not like and what they had learnt.

Nine members of the 20 cast shared their videos uploading a median of 4 (range 1–13) videos, which were between 1 and 18 min long (median 5.03 min). These were reviewed independently by two researchers (RT/LF) who took notes and through comparison of notes and discussion agreed the emergent themes representing their experience of creating the performance. The videos consisted of spoken diaries, raps, and visual drawings of the workshops. Six key themes were identified: initial anxieties; personal development; connections; cancer in young people; impact on the cast; and interacting with researchers.

#### Initial anxieties

This was a different way of working for the current cohort of young people in the CYC compared to other productions they had been involved in. Young people were excited to get started on the new project; however, some of the cast were concerned they had no connectedness to the subject and that it was a difficult subject so questioned how they were going to create a performance that people would want to watch. Others were worried about how it was going to work: *“if we get this wrong it will be worse than anything else”*. The previous production done by the CYC had been a children’s Christmas production so there was a feeling that this was a *“massive transition”* and as it was very academic based on research. Some of the cast felt they were “*out of my depth*” with the topic.

#### Personal development

Many of the cast described their motivation for participating in this performance as an opportunity to develop their skill set as an actor and an opportunity to help break through existing taboo and stigma attached to cancer. *“To just have an all rounded view on cancer. To actually know about it, from patients, carers, from family members, from friends, from doctors, everything. Because obviously we know it kills people but at the end of the day as an actor, I’d like to know about it all so I have that in my pocket as well, so that’s going to be a great experience”*. Some described wanting to be able to understand more about cancer in young people, to raise more awareness and to open discussions that researching cancer in young people is a valuable thing to do and different from adults and children.

#### Connections

Connection to the project and to the group was described. Many of the cast described a nervousness in the beginning due to the topic and the sensitives of working on content of the impact of cancer on young people while having young people with cancer in the cast: ‘*you’re dramatising stories with those people in the room which is a big ask really’* Some of the cast felt they ‘*treaded carefully’* during the first few workshops and felt nervous as the substance was ‘*tricky*’. However, this seemed to be resolved by the third workshop with young people feeling they had built enough bonds to allow themselves permission and “*the right to go forth and produce the show”.* Interestingly, there was a description of using personal experience of anxiety and depression to connect to the cancer experience, after learning that young people often experience anxiety and depression following cancer treatment.

#### Cancer in young people

The cast had very little experience of cancer in young people, but some experience of knowing someone who had cancer. Many were surprised that young people could get cancer and were enthused to learn about it through the workshops. For example, learning about the broad spectrum of research being carried out beyond traditional drug trials. Other key points of learning included the terminology of cancer, i.e. benign tumours are not cancerous, however, cancer can be a tumour, and that there were different types of treatment. The members of the cast who had cancer made the experience real to the cast and some of the experiences *“hit home”*. For example, one of the cast with cancer shared that they were treating to cure her cancer but if the tumour had been 1 cm bigger then it would be a ‘*death sentence’,* because the treatment intention for a 4 cm tumour would be palliative care rather than treating to cure. When discussions arose about the place of care the cast were shocked because they did not realise that some of them would have been placed in a children’s ward depending on age and availablity of specialist services in their area. The cast were also shocked to learn about the long term side effects and wider context of cancer treatment on young people. For example, young people have to considering fertility options such has freezing sperm, eggs or embryos due to the risk of treatment induced inferitilty and early menopause: *‘mind blowing as a 16 year old going through that’*.

#### Impact on the cast

Overall, the cast found the workshop activities insightful and enjoyable. However, there were some instances when the activities had either made them feel uncomfortable (e.g. a body exercise, where the cast needed to identify parts of their body that they were unhappy with) or involved abstract activities that they could not see the relevance to the performance. The cast took time to learn where humour could be used appropriately without people feeling uncomfortable but still highlighting the absurdities of some situations. The cast felt it was important to include humour as ‘*some things in life are funny’.* The strengthening relationships with the ‘*cancer girls’* helped to appropriately facilitate humour into the performance. Members of the CYC who performed in previous productions noted the cast gelled much quicker in this production in comparison to others, probably due to the intense process of dealing with a difficult subject: “Extremely proud of what we developed … So f****** proud about this show. So proud and so glad I got to do this show”.

Both those with cancer and those without described positive effects of being involved, *‘I feel like I am a completely different person to when I started the project, not in a good way and not in a bad way’.* In addition to personal development the cast described learning about a more complete picture of cancer in young people *“just see bits and bobs on TV, ... didn’t really know anything. What I’ve learned in the workshops about cancer and young people falls in line with many things about young people and how we are treated as young people. As society just views us as kids what do they know. The base thing that needs to be acknowledged we are young we are just beginning; this is the most important time*...”

#### Interacting with researchers

Finally, the cast reflected on the involvement of the research and clinical teams who joined the weekly workshops. They enjoyed learning about the reality of being on a hospital ward and having the freedom to ask the researchers/clinicians anything. This was a situation that they did not normally get to experience, only being able to talk to healthcare professionals when they were ill. The cast appreciated that the researchers and clinicians provided clear explanations and *“no question was seen as stupid so felt able to ask them”.* They also enjoyed the range of different perspectives being presented from nurses, medics and researchers.

### Researcher perspective

The research team wrote a reflective diary after attending each of the workshops. Analysis of this showed the power of the activities that were used to relay results to the CYC, which really helped them understand complex data. The research team felt included during the workshops, not just sitting on the periphery to share BRIGHTLIGHT results and how they reflect clinical practice. What was particularly noted by a researcher attending the first then last workshop was the relationship that had developed between the cast but coming in as a researcher for BRIGHTLIGHT they were made to feel part of it despite not attending all workshops.

*“We all got a ‘little bit’ of each workshop experience but it wasn’t until I saw all 5 weeks [in video footage provided by the assistant director] that I can appreciate the enormity of what they have done”*

*“So I left with regret (but also recognising that only so much of us is needed, and not too much), a sense of wonder and above all a confirmation of what a good thing it all was … ”*

## Discussion

We share our experience of using the arts as a meaningful way of knowledge translation. This had a greater impact than we had anticipated; we wanted to increase the reach of BRIGHTLIGHT results and accessibility to audiences which would not typically see and/or understand cancer research results. We reached over 1300 patients, members of the public, third sector representatives and multidisciplinary professionals in 7 months. We attribute this to the Director and Assistant Directors embracing what we wanted to achieve and the Project Director’s passion and experience of the public engagement and cancer. Engaging young people was key as we sought to foster meaningful connections and partnerships to optimise the relevance and impact of the research and hence, made results more accessible to its application into practice [36]. Furthermore, the collective expertise in developing theatre from illness ensured the cast were able to accurately present our research while adding their own interpretations. Their voice was interwoven with our findings and rather than detracting from the research, it added broader context.

We found that theatre enabled complex information to be relayed to healthcare professionals, patients and lay audiences in a way that could be understood, although the fact this came from a programme of research was sometimes not apparent to the audience. The brochure for the performance (Additional file 2) was given freely to the audience and the opening scene provided an explanation of BRIGHTLIGHT, so it is unclear why this was lost in translation. The research team could have provided an introduction; however, this would have potentially detracted from the performance. The performance was timed at a point where we anticipated the final and complete results of the BRIGHTLIGHT programme to be available; however, due to technicalities the results from the cohort [16] were not complete. Thereby, the performance did not answer our overarching question: do specialist services for TYA with cancer add value? If the CYC had developed the script based on the answer to this question it may have been more obvious the performance was derived from research.

The impact of giving young people control over the process (with guidance from the theatre team) could be seen from the benefits to the cast: young people without cancer learnt about cancer in young people; and those without cancer developed confidence in speaking to others about their experience with cancer, and greater acceptance of their cancer. The interesting impact was on the research team: we have extensive experience of collaborating and working with young people [26–28, 31, 37], but this was predominantly led by us. Young people and CYC led *There is a Light* and while we had some anxieties throughout the process which may have been due to the fact we let young people lead and control development of the performance, the respect we have given young people was reciprocated; young people valued the work we were doing and what we were hoping to achieve with our research. They therefore presented what they perceived to be the most important findings. This was very humbling and reaffirms our determination to include young people as core members of the research team because they add an important voice to the process, in particular for interpretation of results.

Developing *There is a Light* was unlike many similar theatrical performances reported in the literature where scripts are developed directly from quotes from qualitative studies [13, 33], directed by the research team [35] or based on published experience [38]. No one way of using theatre can be considered advantageous; the method should reflect the purpose. However, researchers need to be cognisant from the outset the impact the process can have on all those involved, including potential tension between researcher and artistic team [10]. The emotional impact on the cast must also be considered. Contact had experience of providing pastoral care, so this was expected, and suitable processes were in place to support young people. In the reporting of the development of a performance based on metastatic breast cancer, Gray et al. [15] reflected that “the actors were not always prepared for how difficult it was at times to deal with our topic”. Only from this reflection did they suggest a mechanism was needed to support actors. We would recommend ensuring appropriate support is planned from the beginning and readily accessible.

There are limitations with the production of *There is a Light* and our evaluation. The original plan was to have a documentary made alongside the development of *There is Light* and a high-quality video made of the performance. Because the performance was a response to the research, as opposed to a performed report *of* the research, a documentary would have facilitated a process by which the performance is presented with accompanying interviews, statistics, etc., to ensure that the full research context was translated to a remote audience. We were unable to secure a distributor who was willing to commit to airing this so the funding to make the video was withheld. Fortunately, a team of post-graduate students from Salford University filmed and interviewed the cast to develop a documentary about the process and impact of the performance on the cast and audience. The documentary reflected some of the issues reported in our video diaries but also reflected the strength of the relationship that was formed between the cast members (https://vimeo.com/238045094). No ‘official’ recording of *There is a Light* was made but it was streamed live on YouTube on the third performance (https://www.youtube.com/watch?v=j6cXieQeMMQ&feature=youtu.be). We are therefore limited on the extent it can be used as the recording is not of sufficient quality to project in an auditorium, only on a personal computer. Thinking of the legacy of the performance is something that needs to be considered at the onset.

The second limitation was our evaluation of the performance from the perspective of the audience only elicited responses from 15% of the audience. Our response rates were also lower than other reports [15, 32, 35] but it may reflect differences in the way the performance was hosted. *There is a Light* was included as a scheduled performance in three theatre festivals (S!CK, Homegrown and Chrysalis festivals), which the audience had to pay to attend. It could therefore be expected that as part of a night out audience members were reluctant to complete a paper questionnaire about their experience of a social event. A specific question evaluating the performance was not included in the evaluation of the RCN conference so there was limited objective feedback from the nursing perspective. It was included in the evaluation of the patient conference, so we had a good response from young people. However, despite these limitations, our evaluation did capture the impact on the cast as well as the research team, so we have been able to show the benefits and challenges to all parties involved in the process. In retrospect, we should have sought funding for an independent evaluation to assess impact on cast, professionals and audiences.

## Conclusion

Using arts, specifically theatre, as a form of knowledge translation enabled us to share our results to a larger, more diverse audience than traditional scientific/academic methods. The long-term impact of this as a method warrants further investigation. However, for other healthcare researchers who are interested in using theatre we offer the following tips for success:

1. Consider at study inception so it can be embedded in the protocol from the onset.
2. Time the development of the performance when the conclusion of the research is clear, so it becomes the key message.
3. Work with artists who have experience in working with healthcare and/or researchers.
4. Collaborate with a theatre team who have national links so the run of the performance can be extended beyond a single location.
5. From the onset think about how the performance will be evaluated; potentially using more creative methods other than surveys.
6. Think of the legacy – when the production is completed do you want a quality recording so it can be shown to other audiences in the future?
7. Ensure enough funding is secured that covers the cost of developing the production but also the cost of evaluation and developing a legacy.

## Supplementary information

**Additional file 1.**

**Additional file 2.**

## Data Availability

The datasets used in the evaluation of the performance are published in this publication. No additional data are available.

## Abbreviations

CYC: Contact Young Company
FYSOT: ‘Find Your Sense of Tumour’
GP: General Practitioner
NCRI: National Cancer Research Institute
NHS: National Health Service
NICE: National Institute for Health and Care Excellence
NIHR: National Institute for Health research
TYA: Teenage and young adult
YAP: Young Advisory Group
