# Supplementary material for: BRIGHTLIGHT researchers as ‘dramaturgs’: creating There is a Light from complex research data
Source: Res Involv Engagem. 2020 Aug 10;6:48. doi: 10.1186/s40900-020-00222-5 (PMC7418195; doi:10.1186/s40900-020-00222-5)
Supplement: Supplementary file 1 — Additional file 1. [file 40900_2020_222_MOESM1_ESM.pdf]

THERE IS A LIGHT: BRIGHTLIGHT
CYC
MARCH 2017

AUDIO CUE - MOONLIGHT SOUNDTRACK
AV CUE - IMAGES OF THE CAST 'SELFIES' MONTAGE.
AUDIO CUE - END WITH AV - FADE
GRAINNE ENTERS FROM STAGE LEFT TO CENTRE STAGE.

GRAINNE CANCER SONG

GRAINNE SITS DOWN AND PUTS ON A PAIR OF TAP SHOES.
GRAINNE STANDS UP.

GRAINNE: There is a light inside of me,
That you and me both can't see
It glows and glows down below
Why can't cancer fuck off home?

CANCER, you're more annoying than a hot lightbulb
CANCER, can you bulb start burning out

Treatment took off all my hair,
Thank god my boyfriend doesn't care
These wigs are really itchy though
More itchy than crabs down below

CANCER, you're more annoying than a hot lightbulb
CANCER, can your bulb start burning out

Misdiagnosis was the best,
Thank god I don't have IBS
3 year wait was just great
The doc and my insides are Bezzie mates

CANCER, you're more annoying than a hot lightbulb
CANCER, can your bulb start burning out.

GRAINNE CROSS TO STAGE LEFT.
LIGHTS FADE UP
MILLICENT ENTERS TO UPSTAGE RIGHT.
GRAINNE LOOKS AT MILLICENT AND EXITS.

PROTAGONISTS INTRO + 1.

MILICENT: Hi. Hello.

MILLICENT MOVES CENTRE STAGE.

MILLICENT: Buenos Dias!

CIARA: No Millicent!

JOE: (Laughing) Millicent why are you saying 'hi' to the audience? It doesn't make any sense!

JOE ENTERS UPSTAGE RIGHT. CIARA ENTERS UPSTAGE LEFT.

MILLICENT: To connect!

JOE: We don't need to connect! They're here now, they've bought their tickets. Unless they're rude enough to walk out they're trapped! We can say whatever we like to them!

MILLICENT: It's much harder to introduce a show than you think, got any bright ideas?

JOE: Not Hi.

CIARA: Well, Grainne just did a tap routine, why don't we just make it a musical?

MILLICENT: A musical, yeah cool, it's a musical.

CIARA: 5,6,5,6,7,8.

ALL: Welcome to Brightlight, welcome to Brightlight. We're gonna make a show for you. It's about cancer. And about youngsters, we hope you like it, we really do.

MILLICENT GOES OFF ON A TANGENT. MILLICENT GETS CARRIED AWAY UNTIL JOE INTERRUPTS.

JOE: We just need to simplify it I think.

CIARA: It's a show about young people.

JOE: It's about cancer.

MILLICENT: It's about a young person's journey through cancer.

CIARA: What's the Brightlight part?

JOE: Does anyone have a programme?

JOE GETS A PROGRAMME LEAFLET FROM AN AUDIENCE MEMBER

JOE: Brightlight present young patient's perspectives on specialist cancer care in England.

CIARA: So there's tons and tons of brightlight research, and this show is our interpretation of that research.

MILLICENT: Because young people need specific care like the care for kids and then the care for adults. Where's the care for teens? Because teens are at their extremes at that age.I mean, puberty and shit, right?

CIARA: And exams and shit.

JOE: And sex and shit.

MILLICENT: And shit like when you think you're in love and you've found the one and everything's going swell and you know that your children are going to be born a year apart and one's going to be called Theon and one Arya because you both love

game of thrones SO MUCH and you also know that his office and your studio are going to be 15 minutes …

CIARA AND JOE EXIT.

[millicent cont.] …away from your stunning apartment in Manchester city centre so you can both meet up for lunch and get the kids from school and then he dumps you…

EWAN ENTERS WITH CRATE.

[millicent cont.] By text. On your 21st birthday.

EWAN PUTS CRATE DOWN. THE PAIR EXCHANGE LOOKS.
MILLICENT EXITS.

EWAN SOLO

EWAN: I've always been quite interested in astronomy so, I'm gonna talk about that now. The brightest known exploding star is ASASSN-15lh, and it's about 3.8 billion light years away. It was discovered by astronomers in Chile in June 2015. It is about 570 billion times brighter than the sun, making it 20 times brighter than all 100 billion stars in the Milky Way combined. There exists however, even brighter objects. A quasar is the material travelling around a black hole…

NEA, CHLOE, MIRAY, MAILI, AMY V AND SHAQ ENTER TO DOWNSTAGE LEFT, AND BEGIN CIRCLING .

[EWAN CONT]… As it circles the material generates massive amounts of friction as entire stars are pulled inwards resulting in the release of colossal quantities of heat and light. The brightest quasar in the sky is 3c273, in the constellation of Virgo. It is 4 trillion times as bright as our sun, which makes it 100 times brighter than the light of our entire galaxy put together. I think it's quite striking that at the centre of the brightest thing in all existences is the darkest most destructive thing.

SHAQ MOVES UPSTAGE RIGHT.
EWAN EXITS.

CIRCLES OF CANCER

SHAQ: I regret to inform that… We need to talk about… sorry you've got cancer.. You need to come for an appointment. Please bring someone with you.

AMY V MOVES CENTRE

AMY V: It's me, Jane from the office. Don't worry, I've not eaten all of your biscuits! (giggles) I was just wondering when you're coming back, its not the same here without you.….

AMY V: Hiiiiiiiii Julie!! It's my Jane from the office, guess what? Karens still having fish for her lunch so you should be glad your not coming back in! hahha! Anyway, come back, yeah?

AMY V: Hi it's crazy Jane from the office. I've been sending you emails all week. Get back here.

MAILI CROSSES

MAILI: Hey Julie, smiley face. how's it hanging? Do you want to come for a drink? Winky face. Or watch a film? Winky face, winks face. I don't even know what appropriate anymore. I'll just send her a poo emoji.

MIRAY MOVES INTO POSITION DOWNSTAGE LEFT.

CHLOE MOVES INTO POSITION.

MIRAY: I know this isn't how we saw us being together panning out. This kind of things is always happens to someone else somewhere else, but it's not it's happening here to us to you.

CHLOE: Hiya love. Listen, I know this is really hard. But we will get through it. It'll be difficult but we will find a way. I'm so proud of you, you're doing so well. One day we'll look back on this and remember how we stuck together. Try stay positive, it's going to be ok.

NEA: Me? Cancer?

THERE IS A BEAT OF ACKNOWLEDGEMENT.
NEA EXITS STAGE LEFT.
ALL EXIT STAGE RIGHT.

CHAPTER ONE PROTAGONISTS DIAGNOSIS + 5 -

MILLICENT ENTERS
PROJECTOR QUE - 'DIAGNOSIS'

MILLICENT: Diagnosis. Welcome to Channel Four's 'First Dates' where tonight we are at the incomparable Brightdog Ballroom Bar where Joe… JOE…

JOE ENTERS

MILLICENT: …is waiting for his date to arrive. And here she comes, the beautiful CIARA!

CIARA ENTERS

MILLICENT: Oh, aren't they nervous?

JOE: Hi.

CIARA: Hi.

JOE: So, do you want to get some drinks?

CIARA: Yeah, yeah, lets.

JOE: We'll have two Long Island Ice Teas please.

CIARA: (*To audience*) Did he just order for me?

JOE: Can we have extra Coke, don't want to get too pissed.

CIARA: (*To audience*) Is he for real?

JOE: And actually, could you hold the tequila?

CIARA: (*To audience*) Tequila is the best part.

JOE: Maybe skip the triple sec too-

CIARA: Oh my god, why do you keep doing that, why do you keep deciding my order for me?

JOE: I've been here loads of times

CIARA: You have no idea what I want you haven't even asked, I LOVE triple sec (general rant)

JOE: Ciara! Ciara chill!

MILLICENT: There's no real alcohol.

JOE: It's just a scene.

CIARA: Yeah, I know that…this is part of the scene. And I think that I just demonstrated the point pretty clearly. Young people need all the information available to them after diagnosis rather than having healthcare professionals make decisions on their behalf. Don't order my drinks for me Joe!

CIARA EXITS

MILLICENT: She's demonstrated the point to be fair.

MILLICENT EXITS
JOE EXITS. **1 IN 6 SCENE**

AMY V ENTERS FROM STAGE LEFT, TO STAGE LEFT CENTRE.

AMY V: 1 in 2 people globally will be diagnosed with cancer

DAVID enters STAGE RIGHT and crosses to DOWNSTAGE LEFT.

DAVID: No... AFFECTED.

D + A: 1 in 2 people globally will be AFFECTED by cancer.

GRAINNE enters STAGE RIGHT and moves to DOWNSTAGE RIGHT.

GRAINNE: 70% of all cancer deaths are preventable.

MILLICENT enters STAGE LEFT and crosses DOWNSTAGE RIGHT.

MADDIE: No... it's 50%.

MI + MA: 50% of all cancer deaths are preventable.

NEA enters STAGE RIGHT and moves DOWNSTAGE CENTRE.

NEA: 1 in 2 people in the UK will be diagnosed with cancer...

JOSH enters STAGE RIGHT and moves DOWNSTAGE CENTRE.

JOSH: 1 in 2 born AFTER 1960.

NEA: 1 in 2 people born after 1960 in the UK will be diagnosed with cancer.

JOSH: Yeah and 1 in 6 will die of cancer.

PAUSE. ALL EXIT EXCEPT MADDIE.

MADDIE SOLO

MADDIE ENTERS UPSTAGE LEFT. MADDIE BEGINS TO TALK AND THROUGHOUT MOVES AROUND THE STAGE TO HIGHLIGHT MOMENTS IN THE TEXT.

MADDIE: So I'd just come back from the theatre. My front driveway has a mini roundabout in it with grass and trees in the middle. I park to the left of the house just off the roundabout, my dad parks in the middle and my mum parks to the right of the house just in front of the garage. I pulled into my driveway on the 15th of November 2016 at around 11.04 and remember being annoyed that my aunties car was parked in my space. Why was Christine who lives 50 minutes away from my house parked in my driveway? I walked past my dads car to my front door with is bright red and has been ever since we moved to this house. I'd also realised I'd forgotten my key, so rather than wake anyone I decided id break in. It was freezing and my hands were cold. Once I managed to open the door I took my shoes off and put in on the shoe rack to the right of the red door, I walked into the kitchen. The kitchen is to the left of the front door, round an L shaped wall with flower wallpaper on it. The Kitchen is made up of two half's, is all tiled and painted purple. As I walk in, the kettle is on the left of me, the sink is directly in front of the table with windows out to the back garden where the light sometimes flickers on, because we feed the badgers peanut butter and bread each night, and then there's the stove. My mum was stood facing the stove when I asked her what Christine was doing here. She didn't answer my question and just asked me, "do you want a cup of tea mads?" Thats when I knew something was definitely wrong. So I left that room and walked towards my downstairs toilet. I shouted Christine's name and heard some clattering from the downstairs toilet, and by the time I reached the door, she raced past me and into the kitchen. I spun around 180 degrees and followed her into the kitchen. "why are you here?", She didn't answer. So I went to find my dad. I walked back into the main hall way, past the stairs that are opposite the unused room and through into the piano room. That room is usually pitch black at night. But there was a glow from the glass door that leads into the slippy, you call it the lounge, we call it the slippy. I opened the door to see my dad with a pint glass of water just looking at the floor. He was sat in his usual place, of the far right of the centre sofa with the lamp on next to him. I sat next to him in the middle of the crack in the sofa. My mum sat next to me, I don't remember where my auntie went. She explained that shed had an emergency appointment at the hospital that afternoon She told me that she had cancer. She said that it had spread from her bowl to her liver. She told me that there wasn't a cure and that she was going to die, likely within the next three months.

MAILI ENTERS STAGE LEFT.
THEY ACKNOWLEDGE EACH OTHER.
MADDIE EXITS STAGE RIGHT.

AMY'S MIS-DIAGNOSIS SCENE

MAILI MOVES STAGE CENTRE.

MAILI: My name is Maili. This is Amy's story. She really wanted to tell this story to you, but she wasn't here. I have the responsibility of telling her story. I will start at the beginning.

(CHORUS) DUNCAN ENTERS UPSTAGE CENTRE. CHLOE ENTERS CENTRE STAGE LEFT. MIRAY ENTERS CENTRE STAGE RIGHT.

MAILI: Amy worked at carphone warehouse.

CHORUS: Good morning Carphone Warehouse, how can I help?

MAILI: She started feeling sick. Nausea, vomiting and unable to keep food down. So Amy went to the doctors. They began by telling her that she was pregnant.

DUNCAN: You must be pregnant!

CHORUS: We'll check that. It's not that.

CHLOE: It could be Chrones disease?

CHORUS: We'll check that. It's not that.

MIRAY: It could be IBS.

CHORUS: We'll check that. It's not that.

CHLOE: We'll need to send you for a CT Scan.

DUNCAN: We'll send you for an X-Ray.

MIRAY: We need to send you for an MRI.

MAILI: One day Amy was shopping. She received an email from the doctors saying…

CHORUS: We need to have an MDT Meeting.

THE GOOGLE DICTIONARY DEFINITION OF MDT MEETING IS PROJECTED ON SCREEN .

MAILI: Amy didn't know what an MDT MEETING was. So she googled it.

AMY ENTERS CENTRE STAGE.
CHORUS SURROUND AMY FOR TABLAU.
MAILI MOVE CENTRE STAGE RIGHT BEHIND.

MAILI : There Amy was standing on busy high street, reading for first time that she may have cancer.

AMY R ENTERS. DUNCAN, CHLOE, AND MIRAY SURROUND AMY R. TABLAU IS FORMED.

CHLOE: You should have received a letter from the Royal Orthopedic Hospital.

MIRAY: We've received your biopsy back.

CHLOE: You have a tennis ball sized tumour on your spine.

DUNCAN: We need to remove three of your vertebrae.

TABLAU DISSOLVES. CHORUS EXIT.

AMY: My spine.

AMY R TURNS TO LOOK AT THE SCREEN AND THEN BACK TO THE AUDIENCE.

AMY: I woke up after a four hours operation, just shouting for my dad. I was on more drugs than I can name, with wires coming out of places that wires really shouldn't be. i just wanted to get out of hospital. Once they actually find out what was wrong with me, the care I received was really good. But, a year later and my cancer is still the multi-coloured elephant in the room.

AMY R EXITS.

DUNCAN SOLO

DUNCAN ENTERS TO DOWNSTAGE LEFT.

DUNCAN: I have tried, and tried, and tried to write something from my perspective for this piece, but I have really struggled! I feel like I have something to say, but what that is, I don't know. I've spent hours and hours talking to my family about it; and come to the conclusion that I'm having an identity crisis. I mean, even stood here now, I still couldn't tell you whether I had cancer or not! 10 years on! I feel like I'm right on the edge of 'the cancer club'. I don't know whether I'm allowed in or not, yet I don't feel like I fit in with 'normal' people. I'm a shade of grey. But does that even matter? Why am I wasting my time thinking about this? I wish I could just move on, I wish I could just get on with things, but for some reason I can't. And that's my point – I want to say something, but I don't know what! My story is a really positive one. I was only 12 when I was diagnosed with my brain tumour. I don't remember ever being scared. I don't remember ever thinking I was going to die! I just remember someone telling me they were going to get rid of these headaches. In September we'll be 10 years on and I've made, what appears to be, a full recovery. Which is great, but at the same time, other young people continue to fight. Why is it me that's okay!? So yeah – that's my identity crisis. My tumour – cancerous or not? Me – where do I fit in all this? I came here now because I had something to say. Now I've said it. Was it right? I don't know. Does it help? You tell me. Does it matter? Probably not. Time to move on? I hope so!

SOC, BRYONY, EWAN AND KELLY ENTER TO UPSTAGE LEFT TO UPSTAGE CENTRE.
DUNCAN EXITS.

LEFT TESTICLE SCENE

EWAN: The great dragon slayer handsome Paul was slaying dragons one day when he found a lump in his left testicle!

JEN ENTERS STAGE LEFT.

JEN: No, No No, this is someones real story, you can’t completely fictionalise it! He wasn’t slaying dragons!

BRYONY: Lets try comedy then?

EWAN: An avocado

SOK: A golf ball

EWAN: An olive

SOK: A tomato

BRYONY: A tennis ball

KELLY: A bouncy ball

SOK: A vibrating love egg

EWAN: These are all the things I was worried they would replace my left bollock with.

JEN: No, no you can't do that.

KELLY: Fine, if it's truth you want, why don't we just put it out there, in his own words, exactly. 'sorry if it’s a bit long or revealing but pick and choose what you like. In 2014, I think? I found a lump on my left testicle'

JEN: Are you serious – Kelly, do you really think you're the right person to do that?

KELLY: Jen, I'm an actor I can be anything –

BRYONY: To be fair Kelly, if we were casting this you wouldn't be my first choice.

KELLY: Why not?

EWAN: Do you have testicles?

KELLY: No, but I’m just trying to respect Paul’s story by getting it out there.

JEN: I know it’s hard but we’ve got to do it justice.

EWAN: OK, Sok why don’t you try it?

BRYONY, EWAN, KELLY AND JEN EXIT.

SOK: I’m sorry if this is a but revealing. My name is Paul. In 2014 I found a lump on my left testicle. I went to the GP to have it checked out. He said it was a cyst and that it would go away. It didn’t.

SOK EXITS.

AFTER THE SHOCK - PROTAGONISTS 3 + 4

MILLICENT, JOE AND CIARA ENTER.

ALL: After The Shock.

CIARA: After diagnosis young people need to be able to speak about the experience they are going through and they need an accessible platform in order to do that.

MILLICENT: So….like this? this stage, this show, this is our platform.

CIARA: Well we should use it to….

JOE: What THE FUCK DID YOU GUYS DO?! Who did it? WHO FUCKING DID IT?! Brexit? FUCKING BREXIT?! Who voted?? Who DIDN'T vote! Did you? Did you? What about you?! Oh I know exactly which way YOU voted you old bastard! Oooooh, leave the world in tatters for the rest of us will you? Ah great, cheers, thanks a bunch! Where's the fucking pound gone??

JOE DROPS TO THE FLOOR
CIARA AND MILLICENT EXIT.

YOUNG PEOPLE EXCLUDED FROM RESEARCH - GRID SCENE

GRAINNE ENTERS TO UPSTAGE RIGHT.

GRAINNE: Will my treatment affect my ability to work?

CHORUS GROUP ENTER AND SURROUND GRAINNE. THEY BEING SPEAKING (OVERLAPPING EACH OTHER). THE CHORUS DISPERSE AND WALK AROUND THE SPACE IN A GRID FORMATION. GRAINNE CONTINUALLY ASKS THEM QUESTIONS. EVENTUALLY GRAINNE GETS SO FRUSTRATED THAT SHE SHOUTS.

GRAINNE: What type of medication will I be on?

What is Sinacilin?

How long will the chemo session be on for?

How long will I be off work?

Will chemo make me lose my hair?

Will chemo make me lose my hair?

Will chemo make me lose my fucking hair?

THERE IS A SILENCE.

CHORUS EXIT. KELLY ENTERS UPSTAGE RIGHT. GRAINNE CROSSES TO UPSTAGE LEFT. THE PAIR SHARE A LOOK. GRAINNE EXITS. KELLY MOVES CENTRE STAGE.

KELLY'S CANCER STORY

PROJECTOR CUE - WHITE BACKGROUND.

ALL CAST MEMBERS BEHIND GAUZE WALKING AROUND AND CREATING SHADOW PLAY. KELLY TAKES A BREATH. CHORUS

MEMBERS ENTER STAGE SPEAKING AND LEAD KELLY UPSTAGE RIGHT.

PROJECTOR CUE - OFF

FIRST TABLEAUX IS CREATED. (MEDICAL EXAMINATION - DIAGNOSIS).

KELLY: September 2015: The start of the unnatural interest in my cervix.

FIRST TABLEAUX DISSOLVES. CHORUS CROSS STAGE AND LEAD KELLY DOWNSTAGE LEFT. SECOND TABLEAUX IS CREATED. (ISOLATION - LYING DOWN)

CHORUS : TICK, TOCK, TICK, TOCK, TICK, TOCK, TICK, TOCK, TICK, TOCK.

KELLY: Brachytherapy: Isolation for twenty-four hours. My worst fear, a lead encased room, alone with my thoughts with only a scenic display for company. Every so often the lights would flicker in the snow covered cottage, reminding me that it's just digital.

TABLEAUX DISSOLVES.

PROJECTOR CUE - WHITE BACKGROUND

KELLY CROSSES TO CENTRE STAGE. AS KELLY SPEAK CHORUS BEGINS TO EXIT ONE BY ONE.

KELLY: For the tail end of 2015, I was poked and prodded. I was poked and prodded by a doctor. Poked and prodded by a nurse, poked by a doctor and prodded by a nurse. Prodded by a doctor and poked by a nurse. Poked and prodded by myself. And all I wanted through this was my own normal back.

.

KELLY EXITS STAGE LEFT.
AMY V ENTERS TO DOWNSTAGE CENTRE, WITH A MIC.

AMY V STAND UP.

AMY V: So there are two C words . I'm only allowed to say one of them in this show. Hello, I'm Amy, I don't have a stage name. I'm actually trying to get rid of my nicknames at the moment. I've always been the kind of girl to have nick names. When I was young and chubby, it was things like plumper, tubs, fat cancer… that was my mums favourite. I've actually been going to the doctors recently because I've had a bit of a dodgey… Cancer. I hate the doctors, Don't you? so judgmental. They're always like 'do you think maybe you should cut down on your alcohol intake, think about the amount of men your sleeping with and maybe take up some exercise'. Horrible. I swear last time I went in, they were just like, 'still a fat alcoholic slut, ok here's your prescription' and I thought, my god, it's nice that someone finally accepts me for who I am. And another thing they always do is, 'will you just hop on the scales?' No. I will not. I don't weigh myself. I've got a rule. The only way I can tell if I've lost weight is if I don't have to lift my belly up to shave my Cancer anymore. Like I said before the reason I've been to the doctor a lot actually was because of my Cancer. So I had a lot of people looking up there and then the doctors had a look. And she sat me down afterwards she said, 'Amy, it's a very harsh environment up there'. Harsh environment?! Sounds like something off bear grylls doesn't it?! I'm bear

grylls, today I'm inside amys Cancer, only the bravest of men have been up here. Bring your wellies because it can get damp.

AMY V EXITS.

SEX HOMEWORK SCENE

NEA, MADDIE, DAVID AND JOSH ENTER ONE BY ONE ACROSS CENTRE STAGE. EACH FINISHING WITH A SEXY, CONFIDENT POSE. LIGHTS COME UP.
ALL LOOK AT JOSH WITH EXPECTATION.

NEA: Josh, come on, read yours.

JOSH: I didn't think that we would actually have to share it in front of audience.

MADDIE, NEA AND DAVID MOVE TOWARDS JOSH AT CENTRE STAGE.

I mean my grandma's in the audience. I thought we would perform in a creative form! Where's Grainne with her tap shoes.

DAVID: No Josh, this is an important issue. Young people are the only people who have sex so we have to take this seriously.

JOSH: I still don't think that I can.

MADDIE: I suppose it's a bit like this really. Just like I wouldn't want to talk about my sex life infant of this audience, Cancer patients shouldn't have to talk about it to a doctor in front of their mum.

NEA: Yeah true, I mean for example: you! When was the last time you had sex? See they don't want to answer in front of everyone, it's awkward!

DAVID: Well I'm fine when I go to the doctor, I get my bits right out, because you have to don't you.

JOSH: Well go on then read your sex diary.

DAVID: No, my mum doesn't need know about my bits. It's private!

JOSH: That's the point. We've all got to respect…

MADDIE: We've all got to respect that Josh has got to read his diary out!

JOSH: No! We've all got to respect each other's privacy. And even David's privacy, so if he doesn't want to read this out then that's totally fine.

NEA GRABS DAVID'S BOOK. AS MADDIE HOLDS HIM BACK.
NEA STARTS TO READ.

NEA: Dear diary, I don't think that the clitoris exists. I can't find!

DAVID WRIGGLES FREE AND GRABS THE BOOK FROM NEA. DAVID RETURNS TO CENTRE STAGE FOLLOWED BY NEA.

DAVID: That's not true! It was research!

NEA: It's okay, I'll get you a map.

DAVID: Shut up! Now you have to share yours!

NEA: I read one line from yours, that doesn't count!

DAVID: You still read it!

MADDIE: I bet Nea did it in her car.

DAVID: Did you do in McDonalds?

JOSH: I bet you did it Subway didn't you. Did you get a six inch in Subway?

MADDIE: Ooh what about in the cinema!

NEA: Fine!

NEA MOVES DOWNSTAGE RIGHT.

NEA: Dear Diary, Well today made me feel over the moon. Not. I had my third consultation with my regular consultant who I don't get to see that often. The problem is that my mum insists on coming along. Mum I am an adult, I don't need you every second. I love my mum but she needs to let me talk to the consultant about my sex life. I want to know when me and my boyfriend can start having sex again. I don't want her to feel like I don't want her there, I do. Her support means everything to me but as close as we are, I just don't think I can talk about my sex life in front of my mum. I don't want it to seem like I have to discuss something she can't hear though. She will question me no end. I don't know what to do. So, who's next?

ALL EXIT.

NHS VS PRIVATE SCENE

DUNCAN ENTERS TO CENTRE STAGE EATING AN APPLE.
LAURA ENTERS TO CENTRE STAGE.

LAURA: Ooh that looks like a nice apple, is it a pink lady?

DUNCAN: No it's a Granny Smith

LAURA: Ohhh no, pink lady's are much nicer... hey you know what they say... an apple a day keeps the doctor away!

DUNCAN: Hmm, maybe that's why I can never get an appointment with a doctor!

LAURA: Ermmm, no. You can't get an appointment with a doctor because everyone is abusing the NHS!

DUNCAN: Well if we didn't have the NHS then we wouldn't have services like McMillian and teenage cancer wards

LAURA: I'm a healthy person, why should i have to pay for services that I don't need

LAURA AND DUNCAN TURN TO FACE EACH OTHER

AUDIO CUE - WILD WEST

LAURA AND DUCNAN TURN AND WALK TO OPPOSITE SIDES OF THE STAGE.

MADDIE ENTER TO UPSTAGE CENTRE.

MADDIE: Round one!

AUDIO CUE - BELL DING

JOSH ENTERS TO CENTRE STAGE.

JOSH: I I found a lump the other da and i could go into a walk in centre. It did take ages for me to be seen by a doctor though... hmm.

JOSH CROSSES TO STAGE LEFT.

MADDIE: Round two!

AUDIO CUE - BELL DING
AMY R ENTERS TO CENTRE STAGE.

AMY R: Bevan didn't create the NHS just to sell it off to Richard Branson and co. He created the NSH to build a strong and healthy country.

AMY R CROSSES TO STAGE RIGHT.

MADDIE: Round Three!

AUDIO CUE - BELL DING STRAIGHT INTO HOUSES OF COMMONS

ALL TAKE A STEP TOWARDS CENTRE STAGE AND BEING TO TALK OVER EACH OTHER.

JOSH: Brexit means Brexit!

AUDIO CUE - MONKEY NOISES

ALL ON STAGE TO INTO VARIOUS ANIMALS AND EXIT

MADDIE IS THE LAST TO EXIT.

NHS BLIND DATE SCENE

AUDIO CUE - GAME SHOW MUSIC
MANUAL AUDIO FADE WHEN ALL FOUR CHARACTER ARE STANDING ON THEIR BOXES AND BRYONY IS IN PLACE.

BRYONY AND MAILI ENTER TO STAGE LEFT; SHAQUILLE, MIRAY AND LAURA ENTER TO STAGE RIGHT.

BRYONY: Welcome back to NHS Blind Date, and our lovely contestant Maili is getting close to choosing the doctor of her dreams! OK Maili, have you got another question for our dashing doctors?

MAILI: What's so special about your ward?

BRYONY: Doctor number 1, go ahead.

SHAQ: We've got something you can really sink your teeth into – a cup of water for your teeth.

BRYONY: Wow, wow, wow, Doctor number 2, how about you?

MIRAY: Cancer can be scary, so we give you a special snuggly teddy bear when you're on the ward!

BRYONY: Awwww! And Doctor number 3?

LAURA: As a Teenage and young adult specialist We've got specialist facilities and holistic care all in one place!

BRYONY: OK, Maili, what's your next question?

MAILI: If you wanna be my doctor, you've gotta get with my friends – who would you talk to about my treatment?

SHAQ: We'll start up here with the husband, and down here with the children.

BRYONY: It's a family affair with Doctor number 1! Doctor number 2?

MIRAY: Cancer can be scary, so we won't worry you too much with the details – we'll just talk to your parents!

BRYONY: Awwwww! And Doctor number 3?

LAURA: You! And whoever else you want – but that's up to you.

BRYONY: Great stuff! ok Maili, how you feeling?

MAILI: I'm feeling Doctor number 3.!

BRYONY: Doesn't she just? But before we make your match, it's time for – the mystery question!

ALL RUN AROUND FOR A COUPLE OF SECONDS THEN RETURN TO THEIR ORIGINAL POSITION.

BRYONY: Doctors – get ready. Where is your hospital?

DOCTORS ALL HOLD UP A SIGN/PAPER

BRYONY: We have our match!

LAURA STEPS FORWARD

BRYONY: It's Doctor number 1!

LAURA EXITS STAGE RIGHT. SHAQ CELEBRATES AND JOINS MAILI STAGE LEFT.

BRYONY: He's just down the road here in Manchester; Doctor number 2 is in Leeds, while

number 3 is all the way in Glasgow! That wouldn't be very practical would it – long distance relationships rarely work! Don't they make a lovely couple?

MAILI: But I wanted doctor number three!

BRYONY: Join us next week on…

ALL: NHS Blind Date!

ALL EXIT.

AUDIO CUE - GAME SHOW MUSIC

SEXY MELODRAMA CANCER SCENE

JEN ENTERS TO CENTRE STAGE.
AUDIO CUE - MELODRAMA MUSIC
EWAN, AMY V AND SOK ENTER TO CENTRE STAGE.
AUDIO CUE - END

JEN: I did it. I sent the selfie.

SOK, EWAN AND AMY V FALL TO THE FLOOR.

JEN: Dear Diary, so I've been talking to this guy on plenty of fish and he seems really nice but he asked for a selfie. I didn't know whether to send it with my wig on or off, my lips are also really pale and you can see my hicc line eurgh, will I ever feel sexy again?

AUDIO CUE - SEXY MUSIC

SOK, EWAN AND JEN SURROUND AMY V.

AUDIO CUE - END

AMY V: When I was 16 I started having sex with my boyfriend. I wanted to go to the doctors because I was finding sex painful. As I was 16, my mum wanted to come. So I explained in front of my mum that it hurt when I shagged my boyfriend. The doctor told me I probably had an sti and I should go to the clinic.

AUDIO CUE - MAD WORLD

SOK STANDS DOWNSTAGE RIGHT, AMY V AND JEN STAND DOWNSTAGE STAGE LEFT. EWAN FALLS OVER. STANDS UP. DROP A PIECE OF PAPER . PICKS IT UP.

AUDIO CUE - END

EWAN: Dear Diary, today I had my last consultation before I start my course of chemo, during which I was forced into asking the question of how the course would affect my sexual… sex, whilst my mum was sat in the room. For the entirety of the drive home, I talked non-stop about the quality of French cinema, despite only having seen half of Amelie once and literally not giving a flying fuck.

ALL STEP SIMULTANEOUSLY CLICKING THEIR FINGERS, TO DOWN STAGE.

LIGHTING CUE - CENTRE FLORESCENT.

THEY PAUSE.

ALL: (*Whisper*) Cancer.

All EXIT.

PROTAGONISTS - SUPPORT + 2,6,7

CIARA ENTERS.

CIARA: First there's Diagnosis. Then there's After The Shock. Then there's Support. Support. I'm a young person. In a hospital. And I'm sick. (Cough). And I've been on the ward…

MILLICENT ENTERS

M: And we are your environment.

JOE ENTER

M + J: We are your environments.

MILLICENT: Physical.

JOE: Social.

MILLICENT AND JOE START CIRCLING CIARA.

MILLICENT: Physical.

JOE: Social.

MILLICENT: Chillout room.

JOE: Pizza night.

MILLICENT: Videogames.

JOE: Alone time.

MILLICENT: Physical.

JOE: Social.

MILLICENT: Physical.

JOE: Social.

CIARA: Yeah OK we get it. I think we get it, don't we? The physical and social environments are as important to someone as being treated in hospital.

JOE AND MILLICENT AGREE THEN EXIT.
CIARA FINISHES OFF THE PHYSICAL INTERPRETATION OF THE SCENE SHE TRIED TO ESTABLISH AT THE START OF THE SCENE.
CIARA EXITS.

POSITIVE REPRESENTATIONS OF CANCER CHARITIES

CHLOE ENTERS TO CENTRE STAGE LEFT, DUNCAN ENTERS TO CENTRE STAGE AND JEN ENTERS TO CENTRE STAGE RIGHT, EACH SIT ON A CRATE. AMY V ENTER TO BEHIND JEN.

AMY V: This is Jennifer. Jennifer is … How old are you?

JEN: Twenty-two.

AMY V: Jennifer is twenty-two. She lives in Manchester and she has recently been diagnosed with cancer.

KELLY, JOSH AND SOK ENTER TO BEHIND JEN.

KELLY, JOSH, SOK: Awwwwwwwwwww

AMY V: During her treatment Jennifer was very very very depressed. But Luckily for Jennifer she was able to find her love of music through musical therapy classes!

JEN STANDS UP. JOSH, KELLY AND SOK ACT OUT AN AIR BAND AROUND HER AND SHE SINGS MCFLY. AMY CROSSES TO CHLOE.

AMY V: This is Chloe. Chloe is 20. She lives in Newcastle. Chloe was recently diagnosed with Cancer.

KELLY, JOSH, SOK: Awwwwwwwwwww

AMY V: During her treatment Chloe was very very very very very VERY depressed. But luckily for Chloe she was able to find his love for film through movie night!

KELLY, JOSH AND SOK SIT BY THE CHAIR AND CRY.

KELLY: I LOVE my sisters keeper.

JOSH: After this lets watch the fault in our stars!

AMY CROSSES TO DUNCAN.

AMY V: This is Duncan. Duncan is 21. He is based in Cornwall. Duncan was recently diagnosed with cancer.

KELLY, JOSH, SOK: Awwwwwwwwwww

AMY V: During his treatment Duncan was very very very very very very very very very

depressed. But Duncan was able to find his… Love of stamps with his neighbour Keith.

DUNCAN: Stamp collecting?? But I want to play football.

AMY V: Activities not available to everyone due to lack of funding for certain charities in certain areas. Please read terms and conditions before diagnosis.

JOSH: Will you lick the stamp for me?

POSITIVE REPRESENTATION OF CARERS - BARBARA

CIARA, NEA, MILLICENT, SHAQ, AMY R, JOE, MADDIE ENTER TO CENTRE STAGE. AS CIARA BEGINS TO TALK, ENSEMBLE CREATE MOVEMENT AROUND HER.

CIARA: Blood pressure, Research, housework, Uni work, late nights, early mornings, medication, appointments, housework, Uni work, this person that person, this number that number. Blood pressure, Research, housework, Uni work, late nights, early mornings, medication, appointments.

NEA LEAVES THE ENSEMBLE AND STANDS DOWNSTAGE RIGHT AS 'BARBARA'.

CIARA: Barbara. Barbara was my Dad's carer. She was blonde. She always wore her hair in a low ponytail and she always had her navy uniform on too. Before Barbara came it was much more of a struggle. She came before I woke up so I could have extra time in bed, she stayed until he went to bed so that we were both settled for the night. She gave him a shower so that I didn't struggle. Barbara made each day a tiny bit lighter and a tiny bit easier.

CIARA BEGINS TO WALK TOWARDS BARBARA AND ENSEMBLE FOLLOW.

CIARA: No matter what had happened, or how things were worsening, she would come in each morning, open the blinds, and begin chatting away to my Dad as though they were old friends. Thank you Barbara. Thank you.

MILLICENT: For the laughs.

SHAQ: For the smiles

AMY R: For giving me some of my youth back.

JOE: From me.

MADDIE: My dad.

CIARA: and everyone else you went to help that day and every day since. The work you do is incredible and I can honestly say with my hand on my heart that I will never forget it. Thank you. Thank you. Thank you.

ALL EXIT. EXCEPT CIARA, JOE AND MILLICENT - JOE MOVES UPSTAGE LEFT, MILLICENT MOVES CENTRE STAGE.

PROJECTOR CUE - 'DEATH'

PROTAGONISTS DEATH

ALL TAKE A MOMENT TO READ THE SCREEN.

JOE AND CIARA EXIT. MILLICENT CROSSES TO UPSTAGE LEFT.

BALLET DUET

BRYONY ENTERS TO DOWNSTAGE RIGHT. THROUGHOUT MILLICENT SINGS A TUNE.

MILLICENT: Life may try to dim your light, or put it out
But look around and you will be found
You are loved, there is no doubt

BRYONY: Dear Alice, did I ever tell you about Charlotte? She was seventeen when she started getting headaches, and eighteen when she died. Brain tumour. Charlotte loved ballet, and at her funeral someone told us she'd been able to fulfil her dream of going to see the Royal Ballet perform Swan Lake before she died. It was supposed to be an uplifting story, I think, and I don't want to tell anyone how they should feel about their dead loved ones, how they should remember them, but when he said she'd watched the show bent double in pain, I thought it was quite bleak. They put her ballet shoes on top of her coffin, satin ballet shoes and white lilies. I tried to write something about it for the longest time, but in the end there was nothing to say.

MILLICENT: Maybe in time, you will find that it's okay
To not be brave
Cause look around and you are found
You are loved and you are safe

BRYONY: But at least she was loved. She was surrounded by love right up until the day she died, and so are you. I am writing to you now because I love you, and I can't know how much of a comfort that is, but it's a fact, at least. Whatever happens, we'll all try to take care of you. Love, Bryony.

PEOPLE WE HAVE LOST

EACH NIGHT PEOPLE WILL ENTER STAGE INDIVIDUALLY. JOE ENTERS LAST.

GROUP 1	GROUP 2
CIARA	NEA
GRAINNE	STEPH
JOSH	SOK
DAVID	JEN
SHAQ	DUNCAN
MIRAY	AMY V
EWAN	CHLOE
LAURA	AMY R
KELLY	MADDIE
**JOE**	**JOE**

JOE BRIGHTLIGHT SOLO

ONCE JOE HAS ENTERED HE BEGINS HIS POEM. LIGHTBULBS WILL BEGIN TO FLICKER OUT. WITH EACH LIGHTBULB MEMBERS OF THE CAST EXIT.
LEAVING JOE ON STAGE.

JOE: You're not alone, and there is a light,
When the world is dark and uncertain,
There is a light.
Your body decays before the time is right,
It's never right to carry the weight of this burden,
But you're not alone, and there is a light.
White coats bear ill tidings and plight,
Loved ones grieve as your conditions worsen,
But you're not alone, and there is a light.
You do not go gentle into your good night,
When cure is not likely and the end becomes certain,
You rage, rage against the dying of your light.
When all else fails without help in sight,
Alone you approach the call of your curtain.
But you're not alone, and there is a light.
There is a light.

THE LAST LIGHTBULB GOES OUT.
JOE TURNS LOOKS AT SCREEN.

PROTAGONIST SURVIVAL

CIARA & MILLICENT ENTER.

ALL: Survival.

CIARA: 2,405 young people will be diagnosed with cancer this year.

MILLICENT: And what kind of treatment will they get?

CIARA: That depends on where they are in the country.

JOE: What they earn.

MILLICENT: And what specialist care is available in their area.

JOE: We need a standard of care across the board for young people which caters to their individual needs.

CIARA: we need to embrace life … make the most of each an every minute.

JOE: Before this process began, my only experience with cancer had been with people of an older generation. Seeing it from a young person's perspective has made me realise what an interruption to life cancer really is — whatever your age. And it's been truly inspiring to see people my age battle through it and come out on top.

MILLICENT: I think for me, this process has shown me that cancer and terminal illness isn't all about the medical side or the treatment, it really is about people. The experience and journey is so different for every individual no matter their class, race or age, and

that should be kept in mind when providing specialist care. A person is not their cancer.

ALL EXIT.

KELLY SOLO

KELLY ENTERS TO CENTRE STAGE.

PROJECTOR CUE - KELLY'S MEASUREMENTS

KELLY: So I'm going to have chemo, but I'm not gonna lose my hair
And I'm going to have radio, but it's only for a month
And I'm going to have brachytherapy, but they're going to knock me out so I'm not even gonna be there
And they say the tumours 3.8 by 3.9 by 2
3.8 by 3.9 by 2, that's big for a cervix, but they're treating to cure
Stage 2a and its spreading, but they're treating to cure
I'm so tired and I can't function, but they're treating to cure
And they cant find a vein again, but they're treating to cure
And my hip really hurts, but they're treating to cure
And the tumours the size of a pea but…..
The tumours the size of a pea…
They can't see the tumour anymore.

JEN - TATTOO MONOLOUGE

JEN - Everything happens for a reason my mum tells me this all the time. These are lyrics from a band called the dunwells, when I first heard them sing this song these lyrics stuck with me and still do - the only hope of understanding any of this is to take some risks well I'm taking risks. My mum I have matching elephants. My mum is my best friend and although we argue and fight she is always by my side. This tattoo is my Mcfly tattoo I got this when I got my first all clear in to remission, Mcfly have been my favourite band for years and they have also been a massive part of my cancer journey so this is like a thank you to them and a celebration for me getting my all clear - they tell me only the strong survive. My Dimond shining bright and full of colour this tattoo is for my 3 friends who lost their battle to cancer but also helped me through mine I have this to remind me that my friends live through me and that they will always be a part of my life! Becky- so shine bright tonight you and I were beautiful like diamonds in the sky. Mobeen-If I could, then I would I'll go wherever you will go Way up high or down low. Tom- on that morning you were taking from me the angels said they had set you free. My cancer ribbon is now a part of me and my life its orange because that's the colour for leukaemia, me and my dad met Chris martin from Coldplay at the Christie the hospital where I was treated and I got to sing the scientist with him so me and my dad added to our cancer ribbons the lyrics nobody said it was easy its such a shame for us to part. Still got blood I laugh so much at this one I've had it in a place that every time the nurses take my blood and put a cannula in for my 3-monthly bone marrow they will they all read that I still have blood This tattoo also means a lot to me because the band the hunna who's song it is each choose a emoji to represent themselves and to me they are more than just a band they have become my friends Crown represents Jermaine - Devil represents Jack – Rose represents Ryan – Explosion represents – Dan.

AUDIO CUE - THE HUNNA

STEPH'S CANCER STORY / DANCE

AUDIO CUE - STEPH'S MONOLOGUE

STEPH ENTERS STAGE LEFT.

STEPH MAKES HER WAY TO DOWNSTAGE CENTRE.

AUDIO: Let's start with dance. Dance is an expression. I'm not good with words so I let my feet twist and turn. My body is a vessel which I tell my story. Monday- Friday: 5.30am wake up, bus to work, 8.45am take kids to school, 9.30am- teach dance to pre school children, 3pm- pick kids up, 4.30pm- teach dance. Let's talk about cancer, the scans, chemo, blood tests, my veins, steroids, pictures of my insides, stacking books in my face. 'Read this it will explain it all, now go away' let's talk about dance.

AUDIO CUE - DESTINY'S CHILD - LOSE MY BREATH

FIRST GROUP OF CAST ENTER AND START DANCE, AFTER THEY ARE JOINED BY THE FULL CAST.

DANCE ENDS.

ALL: Hit me!

SOME CAST MEMBERS SHOUT 'HIT ME' WITH RESPONSES SUCH AS 'WITH YOU BUDGET CUTS' UNTIL THE FINAL…

ALL: With everything you've got!

LIGHTING CUE - BLACK OUT

END
