# Supplementary material for: BRIGHTLIGHT researchers as ‘dramaturgs’: creating There is a Light from complex research data
Source: Res Involv Engagem. 2020 Aug 10;6:48. doi: 10.1186/s40900-020-00222-5 (PMC7418195; doi:10.1186/s40900-020-00222-5)
Supplement: Supplementary file 2 — Additional file 2. [file 40900_2020_222_MOESM2_ESM.pdf]

CONTACT YOUNG COMPANY

THERE IS A LIGHT: BRIGHTLIGHT

Young people's perspectives on specialist cancer care in England

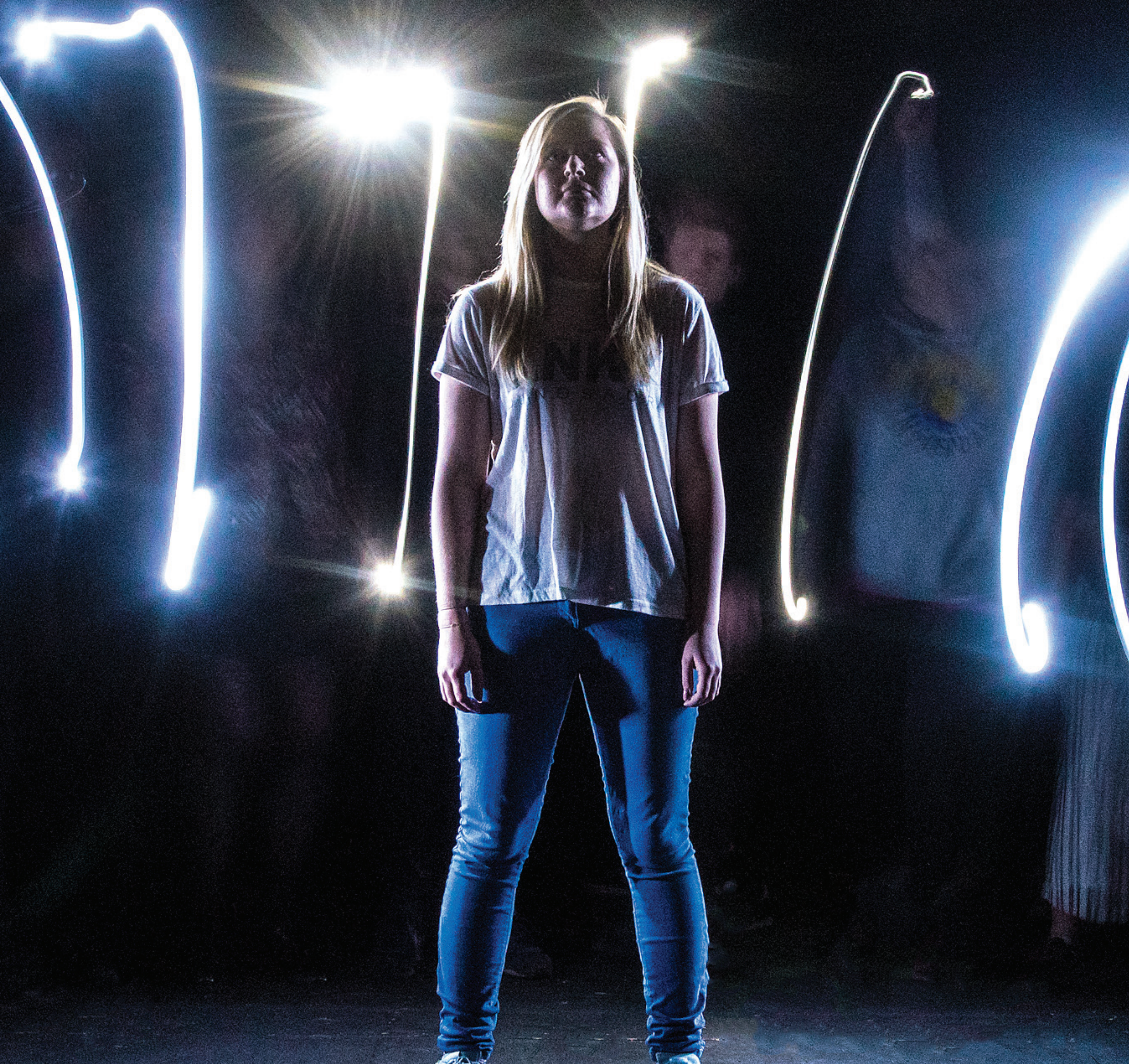

Directed by

Adura Onashile

Developed in collaboration with Brian Lobelcare in England

THERE IS A LIGHT: BRIGHTLIGHT
AN INTRODUCTION

There is a Light: BRIGHTLIGHT is devised in collaboration with the Contact Young Company. It is an attempt to explore the latest findings in a nationwide research programme created by young people. Every year in Britain 2,405 young people will be diagnosed with some form of cancer and yet there is very little care targeted to the specific needs of this age group. Within wider taboos associated with illness in general and cancer in particular, voices of resistance, provocation, humour and empowerment can be drowned out or forgotten. *There is a Light: BRIGHTLIGHT* is our attempt to bring those voices to the forefront. In confounding stereotypes of what cancer and its care means to young people, we hope to shed a light into all the funny, rich, ridiculous, life affirming and challenging stories that also happen to make for compelling theatre.
Adura Onashile
Director

Collaborations between researchers, healthcare professionals and artists are challenging, difficult, time-consuming… and essential. Although we can be mired in the challenges of bringing together such diverse contexts, it is important to remember that such collaboration are also inspiring, reinvigorating, radical, in-depth, and fun. Bringing together the BRIGHTLIGHT research team with Contact Young Company has been a labour of love for all involved: an attempt to translate concepts, contexts, data, agendas and expertise from both directions, with the artistic team grappling with and responding to mounds of findings and data produced by BRIGHTLIGHT over five years, and with the research team grappling with a company's creative response to this same data and findings they produced. As a team, we are proud of not only the premiere of There is a Light: BRIGHTLIGHT, but of the workshops, sessions, phone calls and late night emails between researchers/physicians and performers/artists which have made it the great success it is. Let us hope that these roads of communication and collaboration are now built so that they can be travelled on for years to come.
Brian Lobel

Contact is where young people change their lives through the arts. Based in Manchester, Contact is a vibrant, professional, contemporary theatre and arts venue, with young people driving every aspect of its work. First established as a theatre in 1972, in 1999 Contact reinvented itself as a multi-disciplinary creative space specialising in producing work with, and providing opportunities for, young people aged 13 to 30. Today, Contact is recognised nationally and internationally as a game-changer in the field of youth leadership and creativity. Contact works with the young people of Greater Manchester and with local, national and international artists to create new models of arts engagement – fostering new talent, building skills and ambition – and creating important new productions that tour nationally. Contact is unique in its ethos of placing young people's decision-making at the heart of everything. Young people work alongside staff in deciding the artistic programme, making staff appointments and act as full Board members. This model is seen as a national exemplar of best practice in relation to young people and diversity, influencing organisations nationwide.
Matt Fenton
Artistic Director/Chief Executive, Contact

"What an amazing experience. I was privileged to watch two diverse groups of young people - actors and young people with cancer - together discussing aspects of cancer. They were in turn funny, enlightened, accurate and gave us totally honest accounts of dealing with issues around sex and sexuality as a young person with cancer. I have been working in this field of cancer for 20 years and they managed to make me think about the way in which my team and I work. Powerful stuff!"
Sue Morgan MBE
Teenage Cancer Trust Nurse Consultant. Teenage Cancer Trust Units, Leeds General Infirmary and St James's Hospital

"This really is sharing in research with young people. It's truly inspiring and gives an immediacy of meaning to the research that I couldn't have imagined."
Jeremy Whelan
Professor of Cancer Medicine and Consultant Medical Oncologist The London Sarcoma Service, University College Hospital

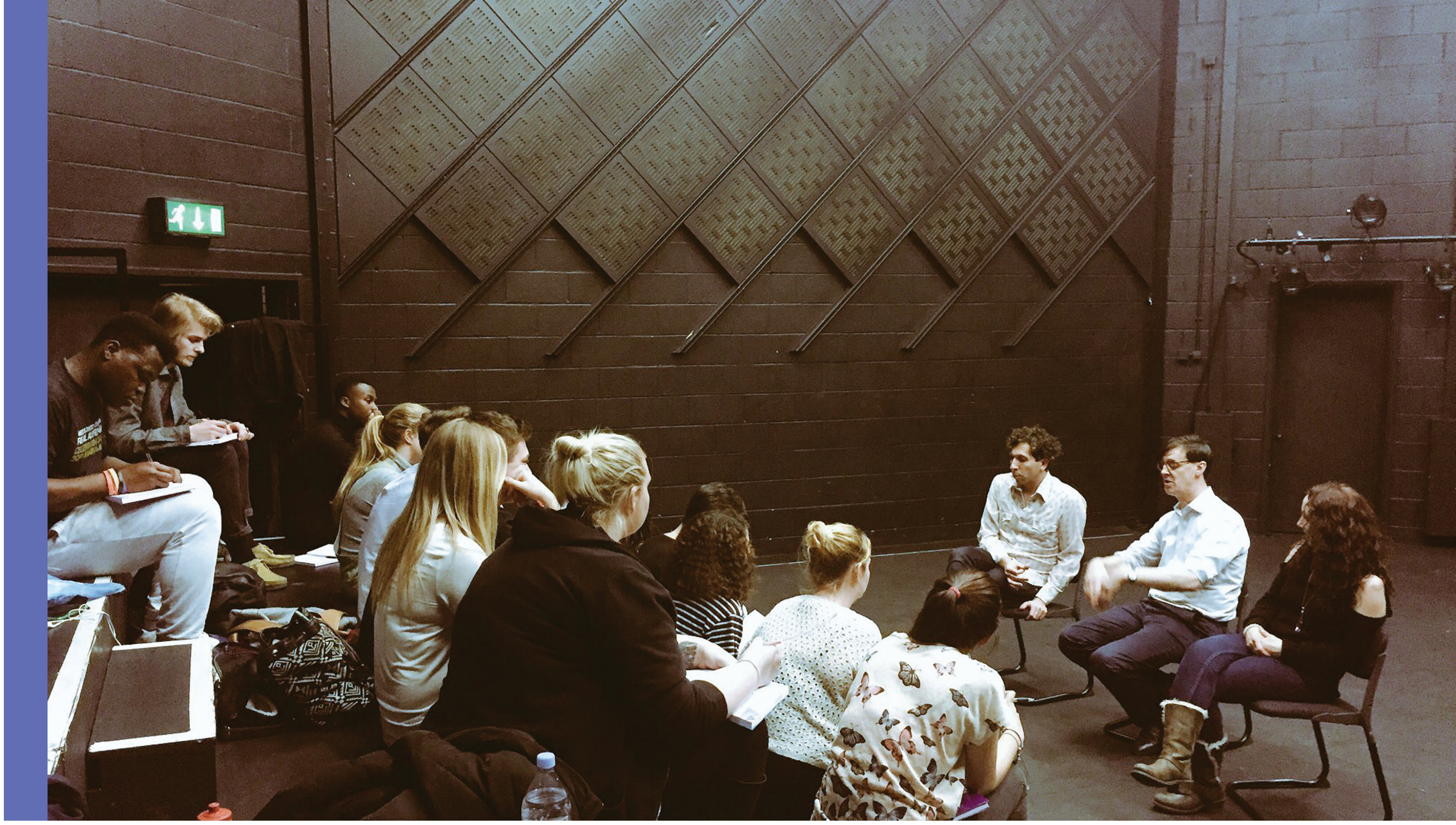

BRIGHTLIGHT
THE STUDY

BRIGHTLIGHT is a National Institute for Health Research funded programme to evaluate specialist teenage and young adult cancer services in England. Young people who develop cancer when they are aged 16-24 years have the opportunity to be treated in one of 13 specialist cancer units across the country or in other centres which are usually more local. These provide high quality cancer care similar to children and older adult cancer units but also provide additional educational, social, employment and peer support.

BRIGHTLIGHT is investigating whether this additional support improved young people's outcome, including quality of life, survival and experience of care. This has been explored in five projects which address the different hospitals young people are treated in, the professionals delivering care, the cost of care and importantly, young people's perceptions and experience of care. 1,126 young people newly diagnosed with cancer between 2012 and 2014 agreed to take part in BRIGHTLIGHT, of whom 830 have completed surveys, which have been administered 5 times over 3 years.

There is a Light: BRIGHTLIGHT represents young people's and researchers' experience of BRIGHTLIGHT.

Take home messages

- When young people get cancer in the UK they are most often cared for by healthcare professionals who are used to looking after younger children or older adults. Young people with cancer need specialist Teenage and Young Adult (TYA) cancer professionals, experienced in focusing on, and communicating with, young people; appropriately involving family and friends; and of course who are also experts in treatments for young people's cancer.
- Distinct spaces in hospital for young people with cancer such as Teenage Cancer Trust Units are more than a luxury. There are good reasons why young people feel out of place in wards designed for children. Specialist physical environments provide an essential focus and platform for professional teams to interact, to give specialist support and for a social environment to exist. This is vital to minimise the impact that cancer has which is to create distance from normal life.
- Supporting young people to 'find' their voice is an important role for a specialist health care team looking after TYA with cancer.
- Special consideration needs to be given to ensure young people have the same opportunities to participate in cancer research as everyone else.
- To achieve the best outcomes for care for young people, we must meet all their needs, which go beyond giving the right chemotherapy or doing the right operation.

THERE IS A LIGHT: BRIGHTLIGHT
THE DEVELOPMENT PROCESS

Joe Clegg
There is a Light: BRIGHTLIGHT has been a uniquely enriching experience for me. From day one we were thrust into the deep end with talk of cancer, what it means to each of us and how BRIGHTLIGHT can directly influence the overall wellbeing of a young person. I knew very little of cancer at the start. I was still under the impression that smoking and other lifestyle choices were the main causes of most cancers! Through guest visits from doctors, patients and researchers from BRIGHTLIGHT, facilitators from the Christie and Brian himself, I've learnt more about the subject in these last few weeks than I ever had. We're also privileged in the way the latest additions to the Young Company have had direct experience with cancer in their past. Their stories have touched each and every one of us whilst providing us all with a really personal insight into what it's like to go through cancer. Each of our thoughts, fears and creative works have been given the utmost support as we toy with what we want this piece to be about. This process has been a unifying experience for us all. Cancer, whilst a dark subject, encourages us to come together as one and I know we'll create something really beautiful from this unity.

Miray Sidhom
The process for There is a Light: BRIGHTLIGHT began with attacking the 'C-word'. The group's relationship with cancer varied from non-existent to personal experience, and everything in between. In the beginning there was a clear distinction between those who had suffered from cancer and those who hadn't, that difference being that we felt unqualified to deal with the topic for fear of sounding ignorant in the process. This was the first challenge, but it was overcome through a series of exercises and workshops that provided us with statistics on young people dealing with cancer, perspectives from nurses and doctors, as well as personal stories from the new members of CYC. In the early stages we tackled the general awkwardness that surrounds cancer by addressing topics that were difficult to talk about, for instance sexual health, fertility and relationships. In doing so, it was evident that when you look at it objectively, we are a group of young people, we have recently undergone that transitory phase of life where our hormones are all over the place and everything seems to be against us. Placing ourselves in that time and trying to imagine what it would have been like to throw cancer into the mix was painfully eye-opening. We then discussed BRIGHTLIGHT as a cancer program designed specifically for young people, and how beneficial it is to have age-specific care when the requirements of a young person are drastically different to those of a child or an adult.

Millicent Thomas
Starting this project was nerve-

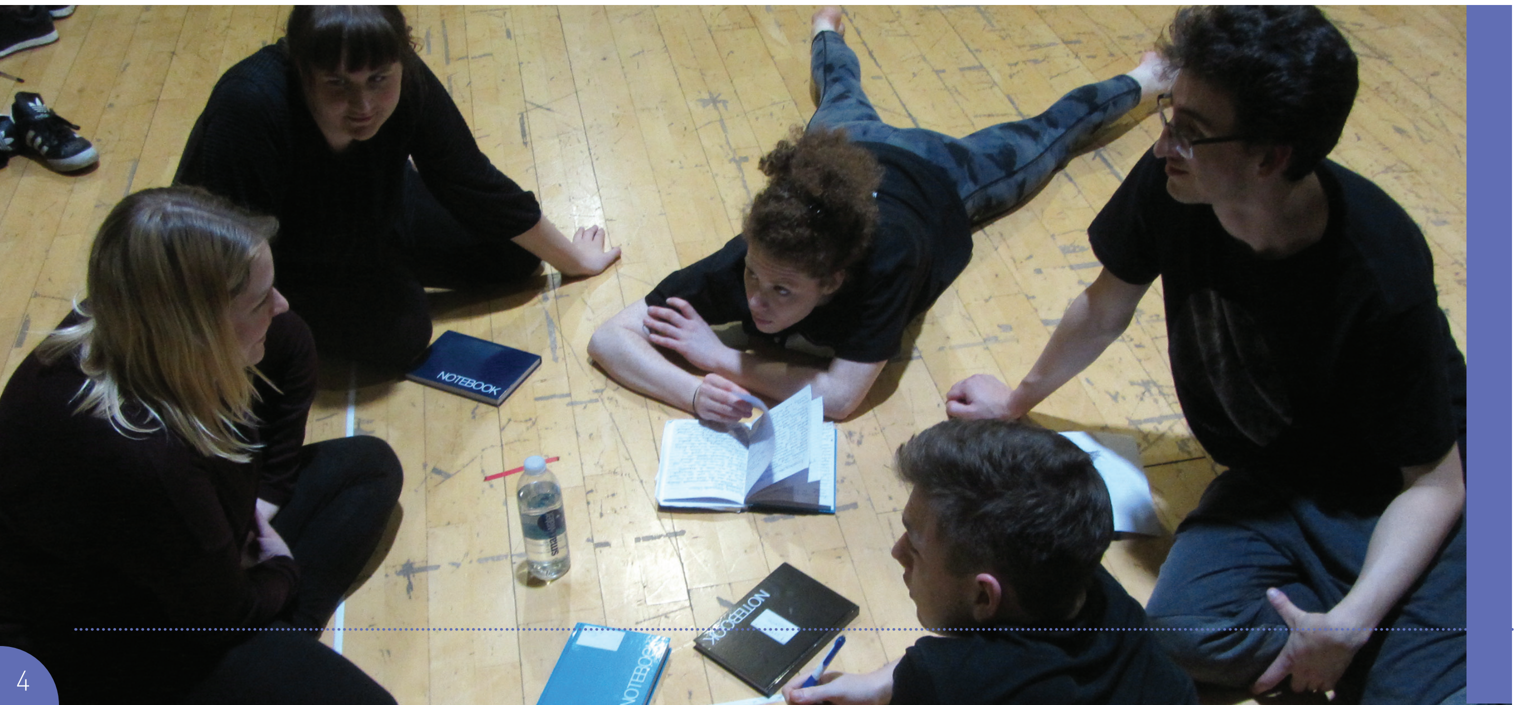

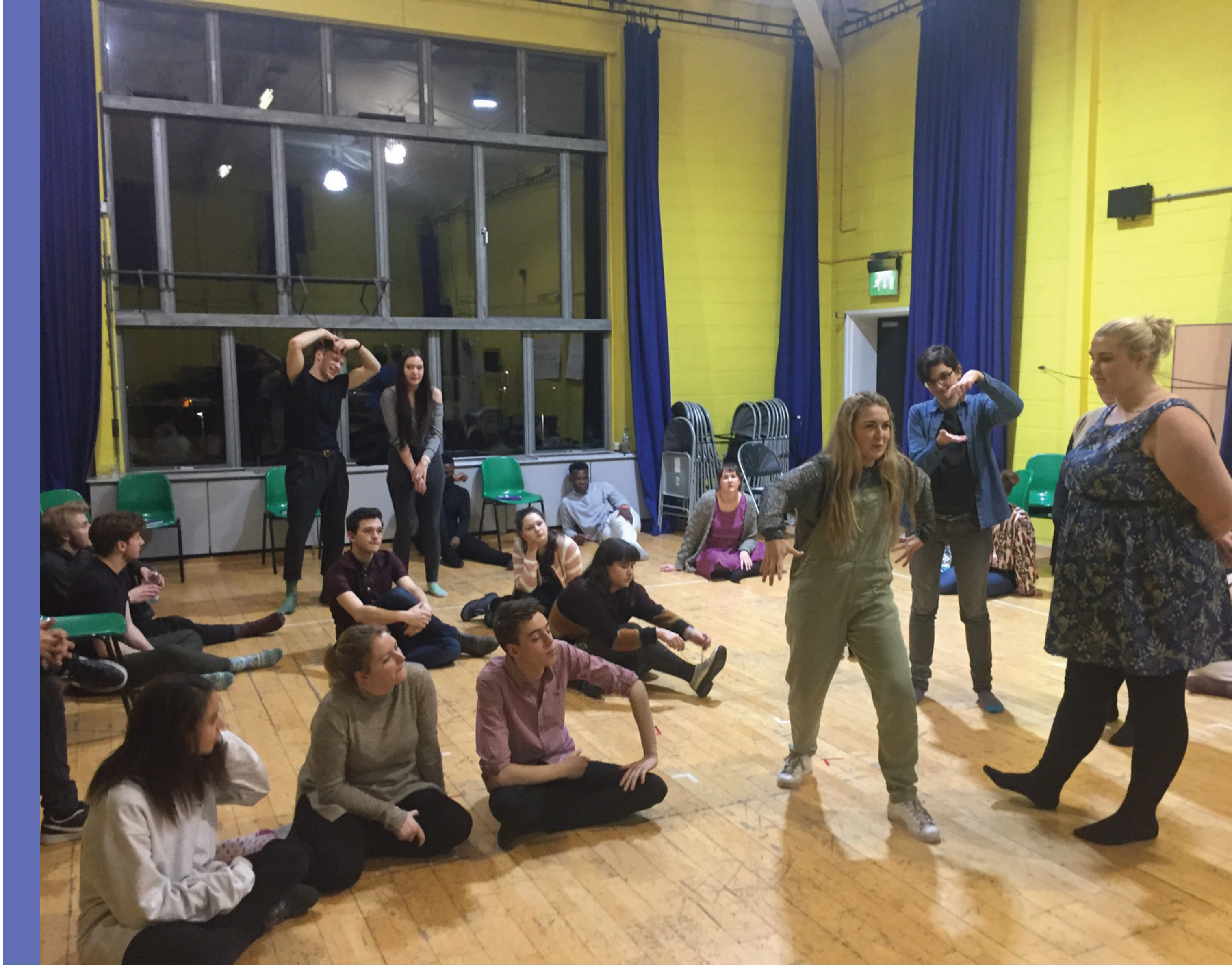

wracking. For those coming straight from the previous show, The Siege of Christmas to this stark contrast in content; and to the new members becoming part of the family (welcomed with open arms, of course). At the start of the workshops there was a lot of trepidation and fear, particularly this fear of 'Do I have the right?' 'Do I have the right to empathise when I can hardly imagine the struggle or process?' 'Do I have the right to ask these questions? Will I offend?' The leadership of Brian Lobel and Nathaniel Hall helped to calm these fears during the workshop period. We took part in many exercises that broke down walls and made us open up completely, becoming much more comfortable asking questions and creating with one another. As a group we have worked hard these past few weeks to create a safe space in which we are free to create, ask questions and be heard without judgement. I am incredibly excited to see the performance develop during these final weeks of rehearsal, I think we're going to make something amazing.

Kelly Pilgrim

I am biased when it comes to the question 'Do specialist services for teenage and young adults with cancer add value?' That is probably best to get out of the way first. This is the question that BRIGHTLIGHT asks, and it is one that I know shaped my own care. The excellent care and support I received afforded me the opportunity and gave me the confidence to rebuild my whole life. I have never been quiet or particularly unsocial, that is quite obvious, but two years ago I would not have stood on a stage and opened up so intimately. When I left hospital and tried to get back to my old life, I was constantly finding that it just wasn't enough. The dull dead end job, the tiny social circle and living for the weekends did not stimulate me anymore. I was offered support from various charities like Climbing Out, Ellen MacArthur and Teenage Cancer Trust and they offered me a way to escape the mundane. Taking risks and doing things that scare me have now been placed as a priority in my life, that is how I have come to be involved in CYC and devising There is a Light: BRIGHTLIGHT. It has been like nothing I've ever done before but it is something I am extremely passionate about because my cancer story, in essence is a success story, however I have met many people for whom it has not been. Getting the personal accounts, the difficult moments and the success stories out there in an innovative way that is not another black and white, forty-three page report with confusing graphs and jarring jargon is an important thing to do and a creative way to reach an new audience. We have been given the means and opportunity to make a change to young adults experience with cancer care. It has been a tiring, heartfelt, stressful (at times) yet healing process creating this piece but we have tried to be true to every person involved and being young people we have that unrivalled ability to speak as young people.

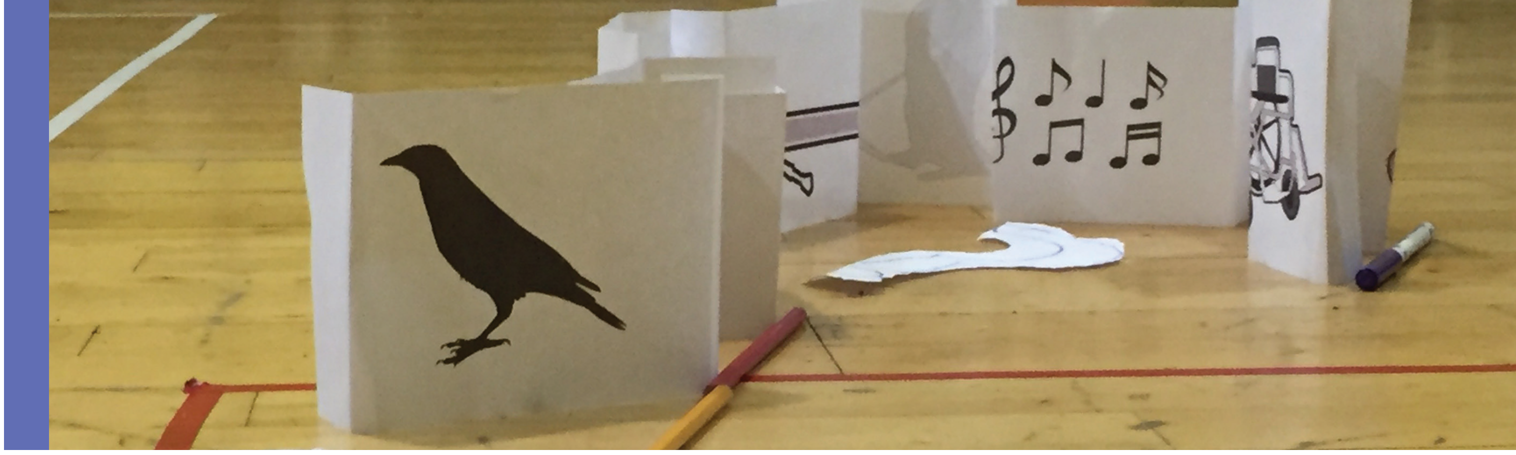

BRIGHTLIGHT
CANCER SERVICES AND YOUNG PEOPLE

But cancer is an old person's disease, isn't it?

Only 1% of all cancer diagnoses in the UK are in young people aged 15-24 years old. While, on the plus side, you have free access to the NHS in the UK, and guidance published in 2005 that states young people need specialist cancer services to make sure you are supported to carry on with your life as 'normal' as possible... on the negative side, you have cancer. You. Have. Cancer. As if being a young person wasn't difficult enough...

Across the UK there are specialist cancer units for young people that provide more than standard treatment. Not only is the decoration in these units more young-person-friendly than a normal hospital ward, but there are specialist professionals available to help you continue with exams, talk to employers, and offer emotional support as well as chemotherapy and treatments. There are also spaces for friends and family to visit. This all sounds good, right? Like a good use of Government health resources, right?

BRIGHTLIGHT is National Institute for Health Research (NIHR) funded research that is asking this question: Do specialist cancer services for teenagers and young adults add value? We want to know if services not only improve young people with cancers chances of survival but also the quality of their survival. BRIGHLIGHT is unique because of how centrally young people were consulted and engaged in the design, recruitment and content of the study, from the name (young people chose BRIGHTLIGHT as they thought the study represented "light at the end of the tunnel, and leading the way for other young people who would go on to be diagnosed with cancer") to the tools by which young people were recruited, with a Young Advisory Panel (YAP) working to get young people involved in the study at the right time, and working to keep patients engaged. Now that BRIGHTLIGHT results are emerging the YAP are helping us develop other research studies.

There is a Light: BRIGHTLIGHT, premiering at SICK! Festival in March is a collaboration between Contact Young Company (one of the country's leading young people's companies, known for their provocative, youth-led projects on pressing topics) and BRIGHTLIGHT, directed by award-winning artist Adura Onashile. Mirroring BRIGHTLIGHT's investment in young people as essential leaders in understanding and treating young adult cancer, the CYC/BRIGHTLIGHT collaboration puts young people in charge of how this ground-breaking research is communicated and disseminated to the public, with a cast of 20 including 4 from Manchester with a personal history of cancer.

After five weeks of intensive workshops with BRIGHTLIGHT's researchers and leads in young people's cancer care – including Sue Morgan MBE, Teenage Cancer Trust Nurse Consultant from Leeds Teaching Hospitals, David Wright Teenage Cancer Trust Lead Nurse from the Christie, Jeremy Whelan, Professor of Cancer Medicine from University College London Hospitals and YAP Member Maria Onasanya - the cast of 20 is now building their own response to BRIGHTLIGHT. Beyond simply a story about an individual's response to cancer, There is a Light: BRIGHTLIGHT looks critically at how young people's voices are included/excluded from research, what cancer means to both people affected physically and those surrounding them, and the larger questions of how we should fund medical care which improves not only physical, but emotional and holistic care.

From the perspective of the research team responsible for BRIGHTLIGHT, the unique collaboration with a young person's company like CYC has been transformative. Confirming the power and intelligence of young people to not only speak up about their own healthcare but also many political, social, economic and spiritual issues as well, the cast's performed and creative interpretations of research have brought critical and unique insights to our own research. The welcoming attitude of the company to our medical research team has also proven, yet again, that collaboration between researchers, patients, families, policy-makers and the public can be enjoyable and bigger than the sum of its individual parts.

We look forward to seeing you at the SICK! Festival and hope you enjoy There is a Light: BRIGHTLIGHT.

This blog/article presents independent research funded by the National Institute for Health Research (NIHR) under its Programme Grants for Applied Research Programme (Grant Reference Number RP-PG-1209-10013). The views expressed are those of the author(s) and not necessarily those of the NHS, the NIHR or the Department of Health.

CONTACT YOUNG COMPANY AND THE BRIGHTLIGHT TEAM

Contact Young Company (CYC) is Contact's award-winning young ensemble, made up of young performers, actors, dancers, musicians, poets and MCs. Each year the company creates brand new professional shows in collaboration with major national and international artists such as Forced Entertainment, Adura Onushile, Slung Low and Grupo XIX de Teatro. Recent successes include Under the Covers, exploring young people and sexuality, which was presented at Contact, Battersea Arts Centre, Southbank Centre and Traverse Theatre. This was followed by a stunning off-site show about the impact of regeneration of communities, *The Shrine of Everyday Things*, which won a Manchester Theatre Award. *The Siege of Christmas,* created by CYC and Slung Low, was a critically acclaimed building-wide adventure for young audiences.

"CYC has opened up so many incredible opportunities that I wouldn't have ever dreamed about a year ago,"
Terri (CYC member)

"Contact Young Company continues to set a precedent for what can be done when young performers are treated as professionals."
The Stage

Cast (in order of appearance)
David Hall, Duncan McCombe, Ewan Orton, Joe Clegg, Joshua Wilkinson, Shaquille Walfall, Sokaribo Dokubo, Tom Durrant, Amy Vreeke, Bryony Bates, Chloe Barlow, Ciara Grant, Gráinne Flynn, Laura Edwards, Madeleine Thomas, Maili Sikk, Millicent Thomas, Miray Sidhom, Nea Edwards-Dixon, Steph Hammersley, Jennifer Miller, Kelly Pilgrim, Amy Riley

CREATIVE TEAM

Adura Onashile
Director
Adura Onashile is a performer, writer and director. Most recently, she wrote and directed Expensive Shit, which premiered at the Edinburgh Festival Fringe 2016. The show won a Fringe First award, the Edinburgh Guide Best Scottish Contribution to Drama 2016, The Scottish Arts Club award. She also wrote and performed HeLa the true story of Henrietta Lacks which won the Scottish Arts Club and Edinburgh Guide Best Scottish Contribution to Drama on the Fringe 2013. Adura has worked as a performer with companies such as National Theatre of Scotland, Untitled Projects, Royal National Theatre, Vox Motus, Royal Shakespeare Company, Young Vic, Theatre Royal Stratford East, Chicago Shakespeare Company and St Anne's Warehouse, New York.

Mark Croasdale, Assistant Director
A multidisciplinary artist and performer, Mark's work includes visual installations right through to immersive drag performance and directing theatre. Mark graduated Contact Young Company himself in 2009, and previously assisted the company on La Bibliotecca.

Nathaniel Hall, Lead Artist
Nathaniel is a proud CYC alumni, who gained notoriety alongside performance artist Stacy Makishi and CYC with Under the Covers, a piece that explored the quest for the female orgasm, provocative diet coke breaks and creative uses for dildos, ball gags and mops. More recently he worked with young people in local schools to develop and perform, Not in My Honour a brand new play about honour based abuse commissioned by Greater Manchester Police and Contact. Nathaniel also works with 20 Stories High and is a Creative Consultant for Contact's Creative Experts.

Keisha Thompson, Producer
Keisha Thompson is a Mancunian writer, performance artist, singer, mathematics tutor and producer who has been loitering in Contact since her teenage days. She has been involved with Contact in a variety of ways including as a member of Young Identity, Re:Con and a Flying Solo participant. Keisha joined Contact as a member of staff in 2011 and is based in the Creative Development office where she is mainly responsible for the award-winning Contact Young Company. She loves engaging with young people and helping them to explore their creative identities.

Aileen Ging, Stage Manager
Aileen has worked at Contact as a technician since being involved in Contact's in-house Technical Theatre workshops, Technique, in September 2014. She currently works as a Freelance Stage Manager and Theatre Technician based in Manchester. She also works in outdoor performance and live events, and has been working with Wild Rumpus as part of their production team since early 2016 on their family festivals and events across the North West.

Jason Crouch
Video Designer
Jason Crouch is a freelance artist and digital consultant, and is currently a PhD candidate at Manchester Metropolitan University. He works extensively with Contact, encouraging and exploring digital innovation. His research investigates digitally mediated intimacy in the modern landscape characterised by the rapid change and widespread adoption of communications technologies. He has worked in theatre and the performing arts for over twenty years; producing large outdoor site-specific work, writing and producing his own plays, providing dramaturgy and technical design for location-specific work.

Dr Rachel Taylor
Senior Research Fellow, Cancer Division, University College London Hospitals NHS Foundation Trust
Rachel is an adult and children's nurse who has specialised in research since 1995. After completing her PhD on teenager's quality of life after liver transplantation in 2008, Rachel joined the teenage and young adult cancer world to help develop the protocol for BRIGHTLIGHT. As well as managing BRIGHTLIGHT, Rachel leads a team of multi-disciplinary researchers conducting studies in a variety of areas focusing on improving young people's outcomes and experiences of care. This includes work funded by Teenage Cancer Trust, the Wellcome Trust, Sarcoma UK and the Bone Cancer Research Trust.

Lorna Fern,
Research and Development Co-ordinator, Teenage and Young Adult Clinical Studies Group, National Cancer Research Institute
Lorna is the Patient and Public Involvement Lead for BRIGHTLIGHT. She has co-ordinated and driven the work of the national group for over nine years. The concept for BRIGHTLIGHT began within the NCRI Group and Lorna worked on the feasibility study Essence of Care and developing the protocol which is now BRIGHTLIGHT. Lorna completed her PhD in Molecular Oncology looking at the long term effects of chemotherapy on the bone marrow. She became interested in young people with cancer while working on an adult bone marrow transplant unit in Nottingham City Hospital. Her other research interests focus on improving access to research for young people and early diagnosis. Outside of work Lorna enjoys yoga and trying to be musical!

WITH THANKS:

Created by Contact Young Company in collaboration with BRIGHTLIGHT, National Institute for Health Research, University College London Hospitals, and University of Chichester.

Commissioned by SICK! Festival

Supported by a Wellcome Trust Arts Award.

WITH SPECIAL THANKS TO:

David Wright | TCT Lead Nurse
Maria Onasanya | Young Advisory Panellist (BRIGHTLIGHT)
Ana Martins | Research Associate
Sue Morgan MBE | TCT Nurse Consultant
Prof Jeremy Whelan | Professor of Cancer Medicine
Kimia Etemadi | Young Consultant (BRIGHTLIGHT)
Natasha Raso | film crew MA students University of Salford
Richard James | film crew MA students University of Salford
Caleb Grinsdale | film crew MA students University of Salford
Ali Obaid | film crew MA students University of Salford

NATIONAL AND LOCAL CANCER SUPPORT CHARITIES:

Climbing Out | climbingout.org.uk | 07977574785
Provide activities to young people facing life changing injury and illness

Teenage Cancer Trust | teenagecancertrust.org | 0207 612 0370
Create world-class cancer services for young people in the UK

Ellen MacArthur Cancer Trust | ellenmacarthurcancertrust.org | 01983 297750
Rebuilding the confidence of young people who have been affected by cancer

Willow Foundation | willowfoundation.org.uk | 01707 259777
Create special days for seriously ill young adults between 16-40 years old

Shine Cancer | shinecancersupport.org | hi@shinecancersupport.org
The only charity that exclusively supports adults in their 20s, 30s and 40s who have experienced cancer diagnosis

CATs (Cancer Awareness in Teenagers) | facebook.com/CATSmanchester
Dedicating to promoting awareness and early diagnosis of cancers in young people

Clic Sargent | clicsargent.org.uk | 0300 330 0803
Support children, young people and families affected by cancer

Anthony Nolan | anthonynolan.org
Support people with blood cancer with particular focus on stem cell donors

Little Princess Trust | littleprincesses.org.uk | 01432 352359
Provide free real hair wigs for girls and boys across the UK & Ireland

Beechwood Cancer Care Centre | beechwoodcancercare.org.uk | 0161 476 0384
Helping people who live with cancer or other life limiting illness

Lime Arts | limeart.org/main.php | 0161 256 4389
Lime is an award-winning arts charity within the Central Manchester University Hospitals NHS Foundation Trust

Francis House | francishouse.org.uk | 0161 434 4118
Francis House provides a home from home where families can receive professional care, support and friendship

Maggie's | maggiescentres.org | 01616 414 848
Maggie's offers free practical, emotional and social support to people with cancer and their families and friends
